# Supplementary material for: Fine-tuning wheat development for the winter to spring transition
Source: Plant Commun. 2025 Sep 5;6(11):101501. doi: 10.1016/j.xplc.2025.101501 (PMC12785163; doi:10.1016/j.xplc.2025.101501)
Supplement: Document S1. Supplemental Figures 1–12 [file mmc1.pdf]

Plant Communications, Volume *6*

Supplemental information

Fine-tuning wheat development for the winter to spring transition

Adam Gauley, India Lacey, Pablo González-Suárez, Harry Taylor, Dominique Hirsz, Sadiye Hayta, Mark Smedley, Wendy Harwood, Simon Griffiths, Shifeng Cheng, and Laura Dixon

SUPPLEMENTARY INFORMATION

Fine tuning wheat development for the winter to spring transition.

Adam Gauley[1, 5], India Lacey[1], Pablo González-Suárez[1,6], Harry Taylor[1,7], Dominique Hirsz[1,4], Sadiye Hayta[2], Mark Smedley[2], Wendy Harwood[2], Simon Griffiths[2], Shifeng Cheng[3, 8], Laura Dixon[1,4]*

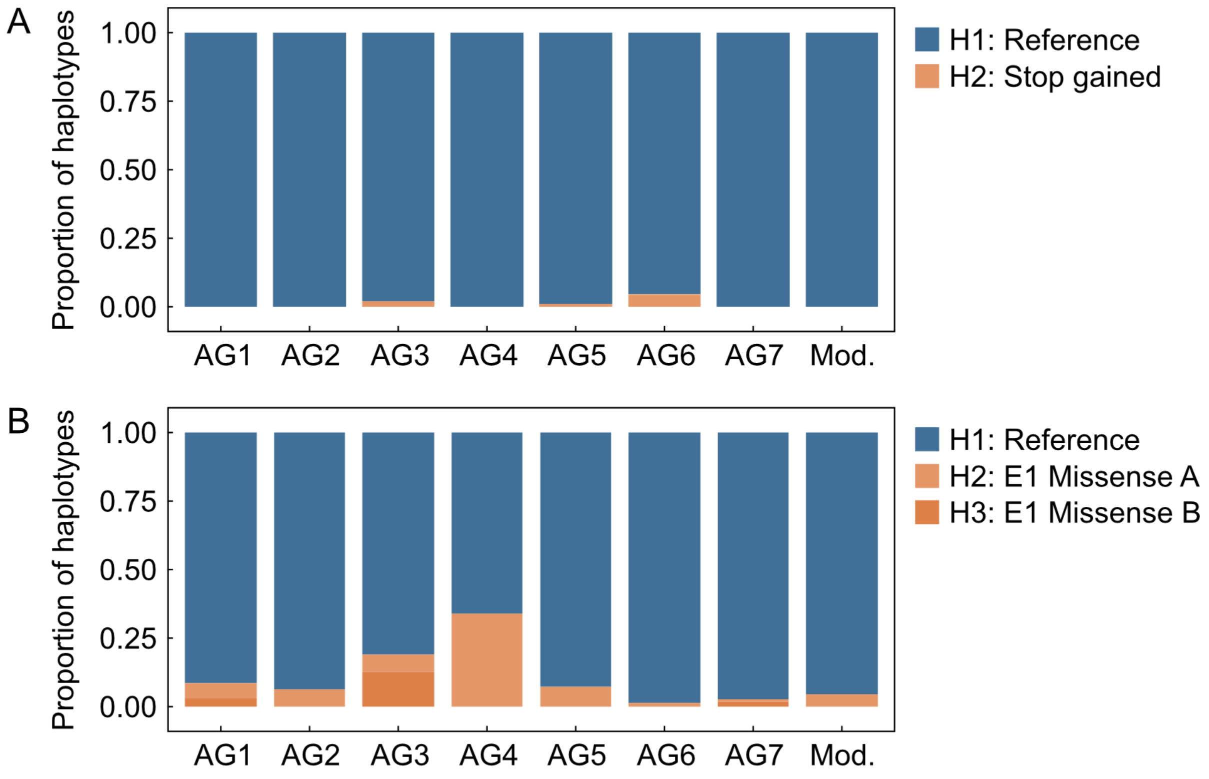

Supplementary Figure S1. Allelic diversity of *FT3* is reduced in the A and D subgenomes

Bar charts showing *FT-A3* haplotypes according to representation in ancestral groups (AG) and modern wheat (Mod.) (**A**). Bar charts showing *FT-D3* haplotypes according to representation in ancestral groups (AG) and modern wheat (Mod.) (**B**). E: exon

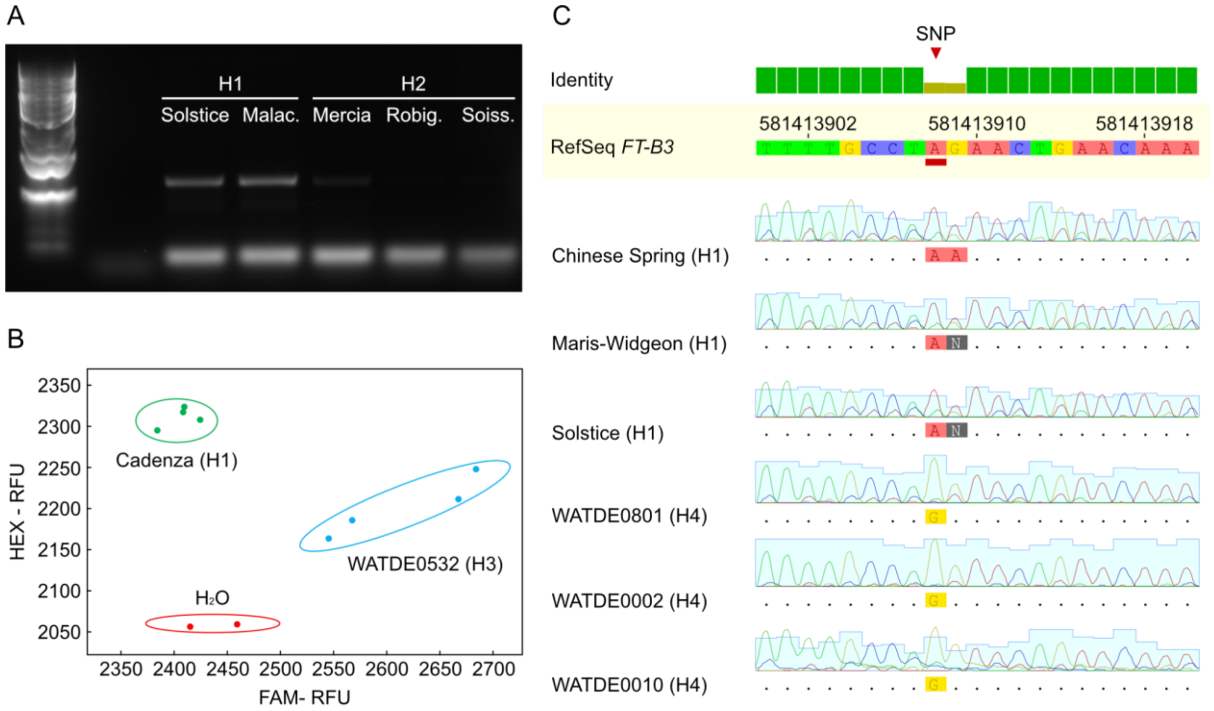

Supplementary Figure S2. Example outputs of markers developed to identify the major *FT-B3* haplotypes

Example 1% agarose gel highlighting expected band for non-deletion versus deleted (H2) *FT-B3* (**A**). Example KASP genotyping output for distinguishing haplotype H3 (**B**). Example sequencing reads for identification of haplotype H4 SNP (**C**). All using primers detailed in **Supplementary Table 2**. H: haplotype

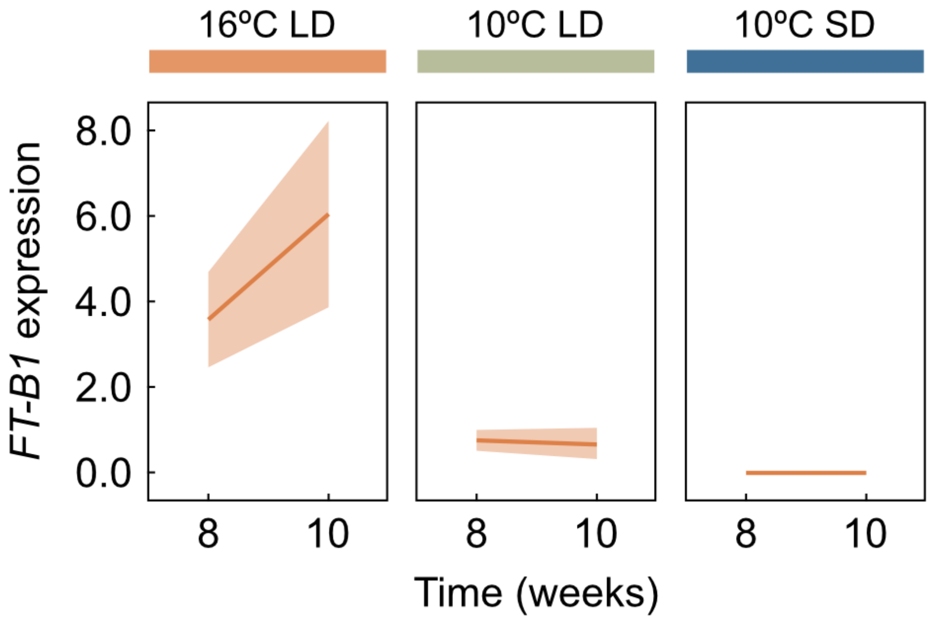

Supplementary Figure S3. *FT-B1* expression across 10-week developmental time-course under varying conditions. As described in **Figure 2F** for *FT-B3* expression (N=3). Lines indicate the average of biological replicates per time point, ribbons indicate standard error of the mean.

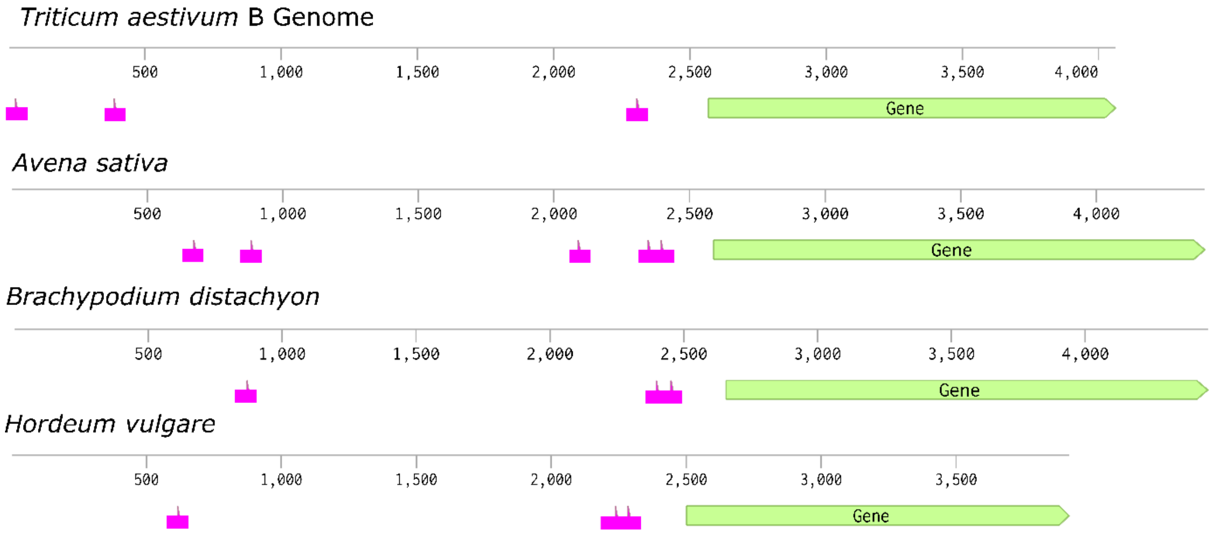

Supplementary Figure S4. MYB binding sites in the *FT-B3* promoter region

FT-B3 gene and promoter map for *Triticum aestivum* and close gene orthologs in *Avena sativa*, *Brachypodium distachyon* and *Hordeum vulgare*. MYB binding domains (AAATATC) within the 2.5KB upstream promoter region are indicated by a magenta box. Gene mapping is indicated in green.

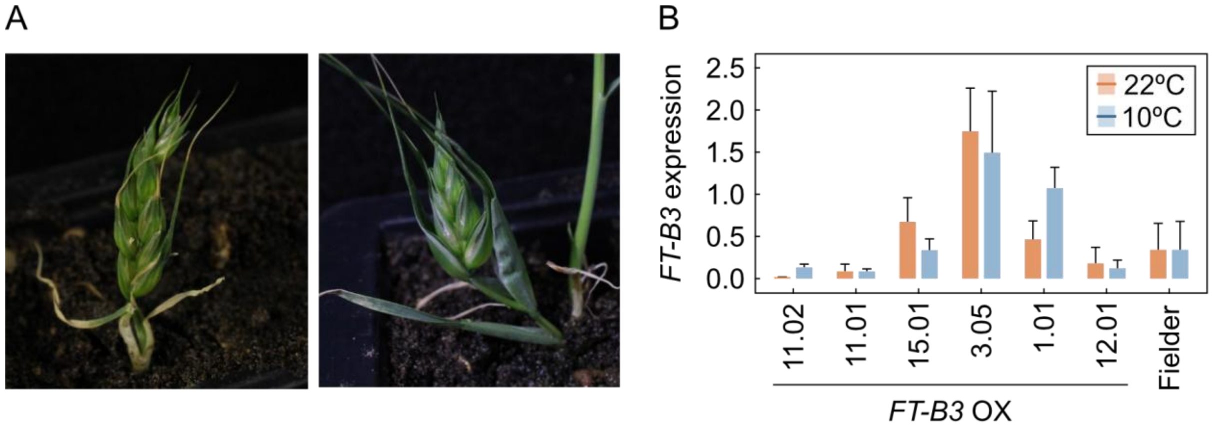

Supplementary Figure S5. Characterization of *FT-B3* overexpression lines

Two example photographs of the exceptionally early flowering phenotype observed in some of the T_0 FT-B3 overexpressing transgenic lines (*pOsAct:FT-B3)* (*cv.* Fielder background) (**A**). Expression of *FT-B3* at 10°C and 22°C in different transgenic lines (N=3) (**B**). Height of bars indicate the average of biological replicates per combination of line and temperature, error bars indicate standard error.

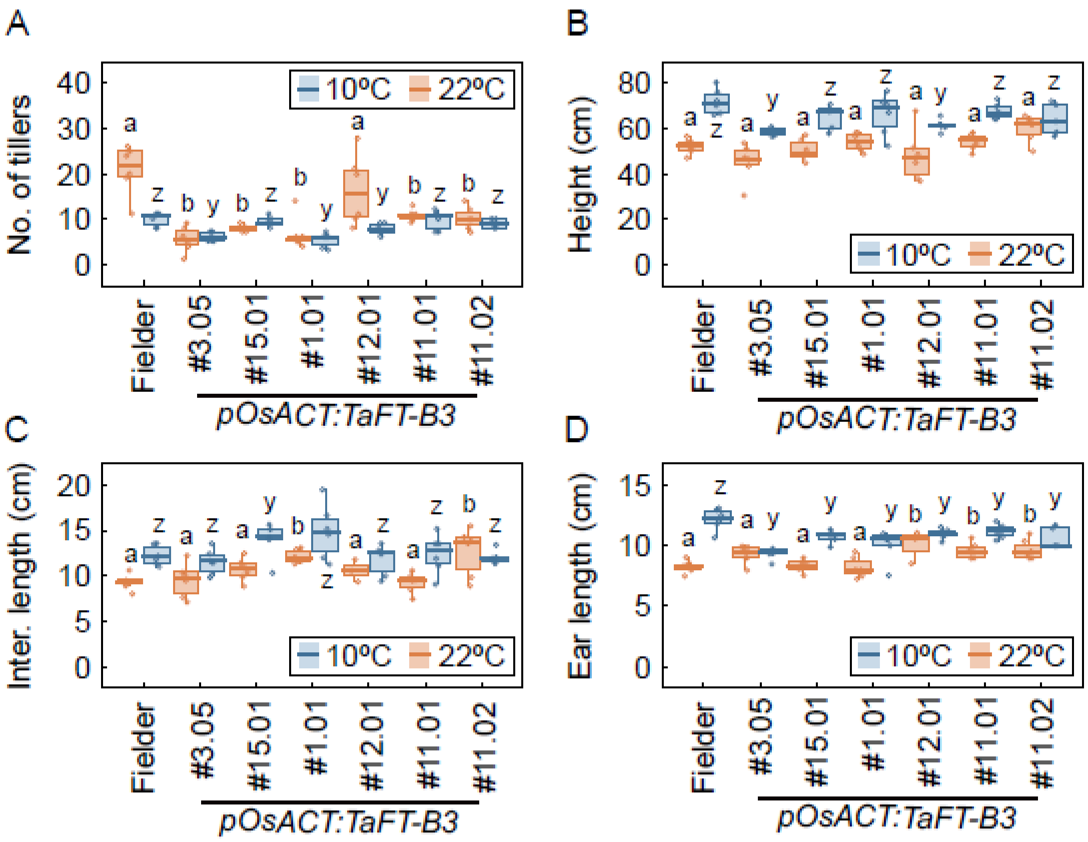

Supplementary Figure S6. Trait characterization of *FT-B3* overexpression lines

Final stage developmental trait measurement for *FT-B3* overexpressing lines (*pOsAct:FT-B3*) at 10°C (blue) and 22°C (orange) ELD (22 h light: 2 h dark). Number of fertile tillers (**A**), plant height (**B**), average final internode (peduncle) length (**C**) and ear length of the first spike (**D**). Different letters indicate statistical differences to *cv.* Fielder within a temperature (ANOVA, Tukey HSD test, $p<0.05$, N=6).

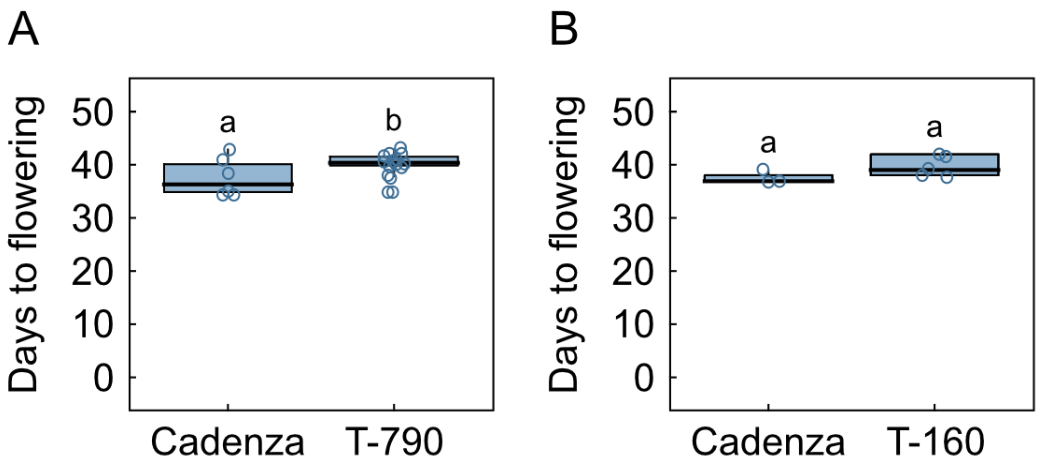

Supplementary Figure S7. Days to flowering of TILLING mutant lines for *FT-B3*

Days to flowering from BC_2F_2 homozygous lines from original TILLING line Cadenza0790 (T-790; *ft-b3*_m1), where different letters indicate statistical differences between lines (Student's t test, $p<0.05$, N=6-14) (**A**) and BC_1F_2 for Cadenza0160 (T-160; *ft-b3*_m2) where different letters indicate statistical differences between lines (Wilcoxon test, $p<0.05$, N=3-5) (**B**) with Cadenza as the background control.

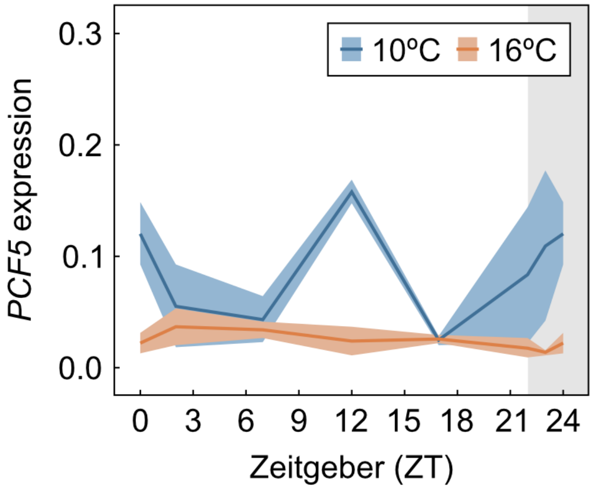

Supplementary Figure S8. Expression of *PCF5* in ELD.

As described in **Figure 3B** for *FT-B3* (N=3). Lines indicate the average of biological replicates per time point, ribbons indicate standard error.

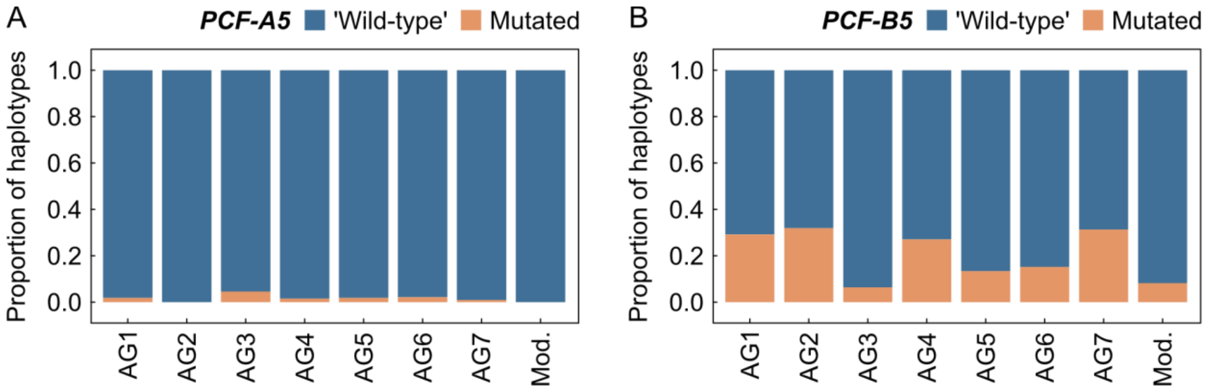

Supplementary Figure S9. Allelic diversity of *PCF5* in the A and B subgenomes

Stacked bar charts showing *PCF-A5* (**A**) and *PCF-B5* (**B**) haplotypes according to representation in ancestral groups (AG) and modern wheat (Mod.)

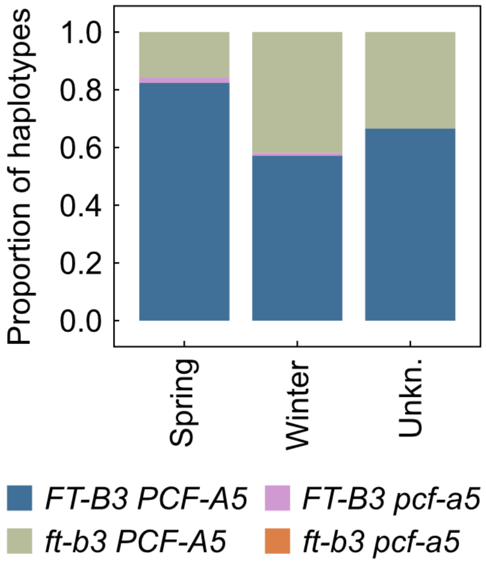

Supplementary Figure S10. Comparison of *PCF-A5* with growth habit and *FT-B3* allele*.* Unkn: Unknown

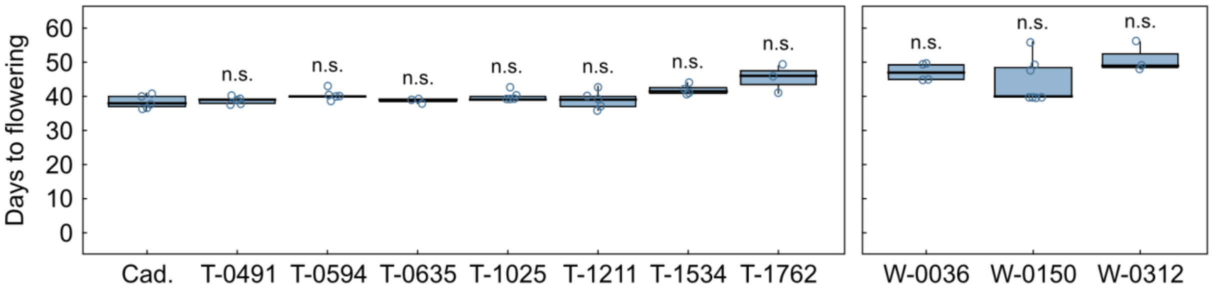

Supplementary Figure S11. Days to flowering for lines with variation in *PCF5* genes

Days to flowering under 22°C ELD of TILLING (T) and Watseq (W) lines with variation in *PCF5* genes, allelic details in **Supplementary Table 5.** Asterisks indicate statistical differences between each genotype and *cv.* Cadenza (Cad.) (Kruskal-Wallis test, Wilcoxon signed-rank test, $p<0.05$, N=3-7), n.s.: not significant.

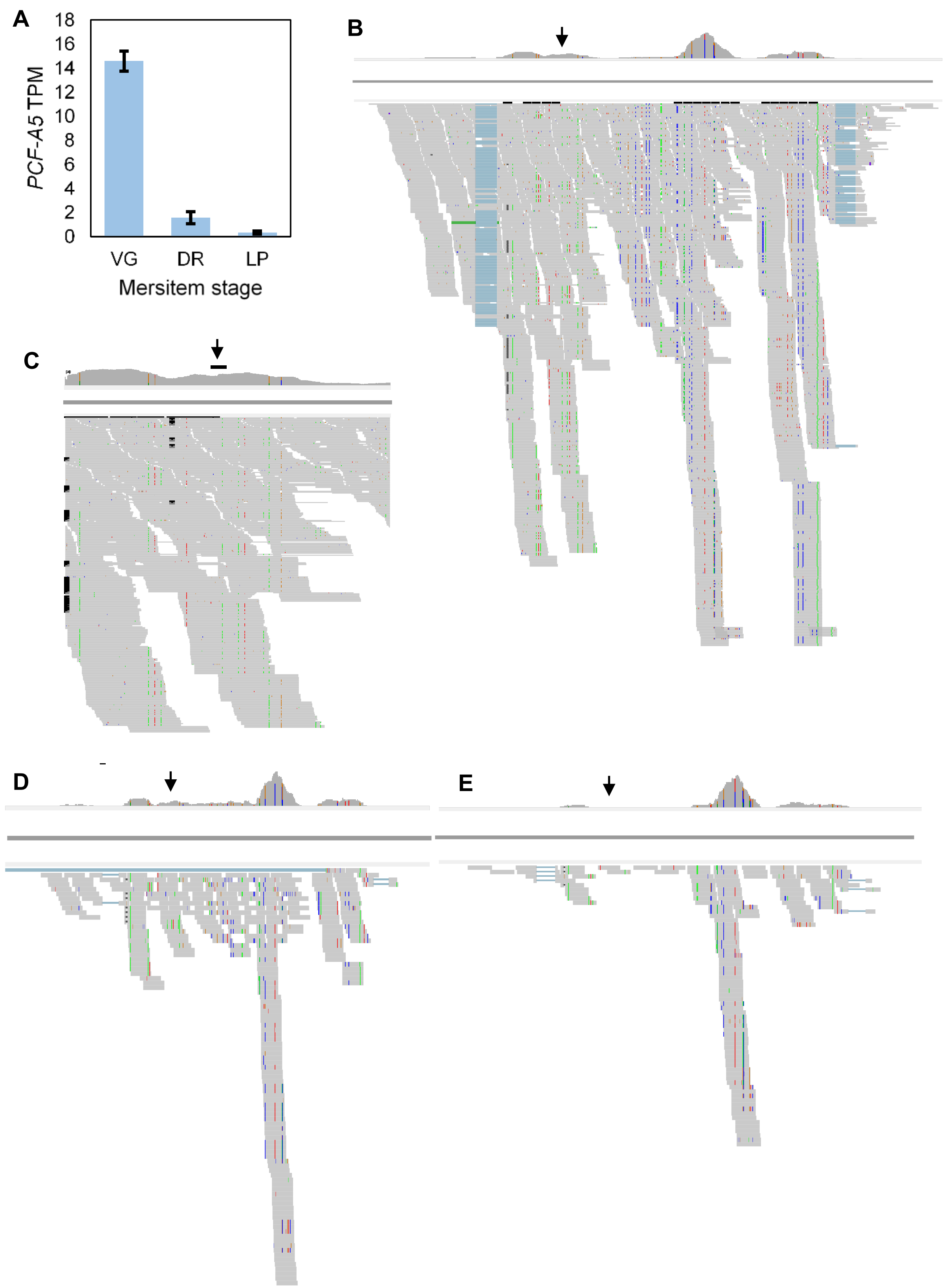
A
B
C
D
E
PCF-A5 TPM
18
16
14
12
10
8
6
4
2
0
VG
DR
LP
Mersitem stage

Supplementary Figure S12. *PCF-A5* expression in the developing floral inflorescence

Transcript profile of *PCF-A5* in the developing floral inflorescence in c.v. Paragon. The defined stages are vegetative (VG), double-ridge (DR), lemma primordium (LP). Data are presented as bar plots that show transcript levels (TPM) ± SEM (error bars) of three biological replicates (**A**). Transcripts aligned to reference genome using IGV (Robinson 2011) to location 3A:119,137,149-119,140,425. MiR319 binding site indicated by a black arrow (**B-E**). Transcripts aligned to VG stage (**B**). Enhanced view of MiR319 binding site for VG stage (**C**). Transcripts aligned to DR stage (**D**). Transcripts aligned to LP stage (**E**). RNA-sequencing data obtained from (Gauley et al. 2024).

SUPPLEMENTARY TABLES

Supplementary Table 1. Genomic variation in FTB3 haplotypes relative to the reference genome Chinese spring.

Supplementary Table 2. Haplotype primers for FT3

Supplementary Table 3. Primers used in this study

Supplementary Table 4. Germplasm used in this study

Supplementary Table 5. Genomic variation in PCF5 haplotypes relative to the reference genome Chinese spring.

References for Supplementary Information

Gauley A, Pasquariello M, Yoshikawa GV, Alabdullah AK, Hayta S, Smedley MA, Dixon LE, Boden SA. 2024. Photoperiod-1 regulates the wheat inflorescence transcriptome to influence spikelet architecture and flowering time. *Curr Biol* **34**: 2330-2343 e2334.

Robinson JT, Thorvaldsdóttir, H., Winckler, W., Guttman, M., Lander, E. S., Getz, G., and Mesirov, J. P. 2011. Integrative genomics viewer. *Nature Biotechnology* **29**.
