# Supplementary material for: Fine-tuning wheat development for the winter to spring transition
Source: Plant Commun. 2025 Sep 5;6(11):101501. doi: 10.1016/j.xplc.2025.101501 (PMC12785163; doi:10.1016/j.xplc.2025.101501)
Supplement: Document S2. Article plus supplemental information [file mmc3.pdf]

Plant Communications
Research article

Fine-tuning wheat development for the winter to spring transition

Adam Gauley[1,5], India Lacey[1], Pablo González-Suárez[1,6], Harry Taylor[1,7], Dominique Hirsz[1,4], Sadiye Hayta[2], Mark Smedley[2], Wendy Harwood[2], Simon Griffiths[2], Shifeng Cheng[3,8] and Laura Dixon[1,4,*]

[1]School of Biology, Faculty of Biological Sciences, University of Leeds, Leeds LS2 9JT, UK

[2]Crop Genetics, John Innes Centre, Norwich Research Park, Norwich NR4 7UH, UK

[3]Agricultural Genomics Institute at Shenzhen, Chinese Academy of Agricultural Sciences, Shenzhen, 518120, China

[4]Leibniz Institute of Plant Genetics and Crop Plant Research (IPK), Gatersleben, D-06466, Seeland, Germany

[5]AFBI, 50 Houston Road, Castlereagh, Belfast BT6 9SH, UK

[6]Department of Developmental Genetics, Centre for Plant Molecular Biology (ZMBP), Eberhard Karls University, D-72076 Tuebingen, Germany

[7]Department of Plant Sciences, University of Cambridge, Downing Street, Cambridge CB2 3EA, UK

[8]School of Biological Sciences, University of Hong Kong, Pokfulam, Hong Kong, China

***Correspondence: Laura Dixon (dixon@ipk-gatersleben.de)**

https://doi.org/10.1016/j.xplc.2025.101501

ABSTRACT

The coordination of floral developmental stages with the environment is important for reproductive success and optimization of crop yields. The timing of different developmental stages contributes to final yield potential, with optimal adaptation enabling development to proceed without being impacted by seasonal weather events, including frosts or end-of-season drought. Here, we characterize the role of *FLOWERING LOCUS T 3* (*FT3*) in hexaploid bread wheat (*Triticum aestivum*) during the early stages of floral development. By assaying the genetic diversity of landraces and modern wheat varieties, we identified a distribution of alleles for *FT3* that indicated selection in modern varieties. We generated transgenic overexpression lines and found that FT3 is as powerful a florigen as FT1, which suggested that *FT3* is under tight regulation. To investigate this possibility, we measured *FT3* expression under variable environmental conditions and identified a role for both temperature and photoperiod in *FT3* regulation. Gene expression analysis showed that *FT3* transcription is partly coordinated by a temperature-sensitive pathway consisting of a TEOSINTE BRANCHED 1–CYCLOIDEA–PROLIFERATING CELL FACTOR (TCP) transcription factor and a warm-temperature-responsive microRNA. We show that this regulation is important for the timing of floral development under short days combined with lower ambient temperatures and that there has been strong selection on *FT3* during cultivation. Deploying this understanding to enable targeted combinations of alleles involved in adaptation will further our ability to develop climate-change-robust cultivars.

Key words: bread wheat, temperature, floral development, FT3, photoperiod

Gauley A., Lacey I., González-Suárez P., Taylor H., Hirsz D., Hayta S., Smedley M., Harwood W., Griffiths S., Cheng S., and Dixon L. (2025). Fine-tuning wheat development for the winter to spring transition. Plant Comm. **6**, 101501.

INTRODUCTION

Crops, like all plants, sense and anticipate changes in the environment to coordinate development under optimal conditions. The genetic regions that control this ability have been under strong selection during crop improvement and are significant contributors to higher yield potential. In the major global crop allohexaploid bread wheat (*Triticum aestivum*), several genes have been pivotal in adapting the environmentally sensitive process of flowering to different climates; these include *PHOTOPERIOD 1* (*Ppd-1*) and *VERNALIZATION 1* and *2* (*VRN1* and *VRN2*) (Worland et al., 1998; Yan et al., 2003, 2004). Inadvertent early selection on these genes enabled the requirements for long-day (LD) photoperiods (*Ppd-1*) and a prolonged period of low temperature (*VRN1* and *VRN2*) to be altered and so propelled wheat into global cultivation. Further molecular characterization of these pathways has shown that they both converge to promote the expression of *FLOWERING LOCUS T-LIKE 1* (*FT1*, or *VRN3*)

© 2025 The Authors. Published by Elsevier Inc. on behalf of CAS Center for Excellence in Molecular Plant Sciences, Chinese Academy of Sciences, and Chinese Society for Plant Biology.
This is an open access article under the CC BY license (http://creativecommons.org/licenses/by/4.0/).

(Yan et al., 2006). Orthologs of *FT1* have been studied extensively in rice (*Oryza sativa*) and *Arabidopsis thaliana* and are a key signal for coordinating floral development with the environment in these species (Yamamoto et al., 1998; Jaeger and Wigge, 2007). Unlike many genes that exert their biological effects in the cell where they are synthesized, the FT protein product is mobile (Tamaki et al., 2007; Chen et al., 2018). It is synthesized in the leaf phloem companion cells and transported to the apical meristem, where it triggers the vegetative-to-floral transition (Chen et al., 2018).

In bread wheat and the close diploid relative barley (*Hordeum vulgare*), there has been a considerable expansion of the *FT* gene family, with 12 *FT* orthologs identified (Halliwell et al., 2016; Bennett and Dixon, 2021). The roles of *FT1*, *FT2*, *FT3*, and *FT4* have started to be explored and show intricate neofunctionalization (Casao et al., 2011; Zikhali et al., 2017; Mulki et al., 2018; Shaw et al., 2019; Gauley and Boden, 2021; Pieper et al., 2021). *FT1* acts most similarly to *Arabidopsis FT* as an LD florigen, showing higher expression under warm, LD conditions (Yan et al., 2006). In wheat, overexpression of *FT1* leads to rapid early flowering, demonstrating its role as a floral promoter (Lv et al., 2014). Unlike *FT1*, *FT2* is expressed in the inflorescence during the lemma primordium to terminal spikelet transition and is a key regulator of spikelet development (Gauley and Boden, 2021). This contrasts with *FT3*, which, like *FT1*, is expressed in the leaf but under short days (SDs) and has a role in promoting floral development under conditions not traditionally considered floral inductive (Laurie et al., 1995; Faure et al., 2007). In barley, *HvFT3* has been under active selection in modern cultivars, with the recessive non-functional allele of *FT3* being most prevalent in northern European winter barley and the functional *FT3* alleles being prevalent in spring and winter barley from southern Europe (Kikuchi et al., 2009; Casao et al., 2011). In the southern European environment, *FT3* is predicted to promote floral development in non- or partly vernalized winter crops under SDs (Casao et al., 2011). Supporting this hypothesis, a role for barley *FT3* in regulating the vegetative-to-reproductive developmental transition has been identified (Mulki et al., 2018). Overexpression of *FT3* caused photoperiod-independent acceleration of spikelet primordium initiation but did not accelerate floral development (Mulki et al., 2018). In bread wheat, the absence of this gene has been linked to important yield-component factors, promoting yields between 3.8% and 7.6% in spring wheat cultivars (Dreisigacker et al., 2021). This not only demonstrates that the environmental regulation of early apex development is distinct from that of heading but also highlights the hitherto largely missed opportunity for utilizing this dual regulation to improve cereal adaptation and final yield potential.

How *FT* genes are involved in modulating floral developmental responses is starting to be understood. FT indirectly associates with DNA through the formation of protein complexes with 14-3-3 and FD proteins to form the floral activating complex (Abe et al., 2005; Wigge et al., 2005; Taoka et al., 2011; Li et al., 2015). In wheat, the specificity of the interaction between FD-like (FDL), 14-3-3, and FT1 has been explored through yeast-based assays (Li et al., 2015). In addition, FTs form other, presumably regulatory, protein complexes (Aguilar-Martinez et al., 2007), the most notable of which involve interactions with the TEOSINTE BRANCHED 1 (TB1)–CYCLOIDEA–PROLIFERATING CELL FACTOR (TCP) family. Within the TCP family, FT-specific interactions have been observed, some of which are conserved between plant species. For example, an FT–TCP interaction is observed in *Arabidopsis* (FT–BRC1) and between the corresponding orthologs in wheat (FT1–TB1), but the signaling outcome is different owing to differences in plant morphology (Niwa et al., 2013; Dixon et al., 2018). Identifying protein interaction partners of FTs and their biological implications offers a clear route to regulating plant development.

Given the known roles of *FT* and *FT*-like genes in the integration of environmental signals, understanding how this integration occurs under realistic conditions is becoming paramount. In northern Europe, the initial stages of floral development in cereals take place during relatively mild-temperature, post-winter conditions, which are suboptimal for stem elongation and flower emergence. Therefore, to understand these early developmental stages, studies need to be performed at lower ambient temperatures than those classically used in controlled chamber experiments. Considering this and building upon the research conducted in wheat and barley identifying *FT3* as an SD florigen, we used realistic conditions based on field temperature data from Norwich (UK) to investigate the role of *FT3* during the early floral developmental period in bread wheat. In this study, we aimed to understand the role of *FT3* independent of vernalization-induced floral development, to determine whether it integrates environmental signals, and to establish whether it has a distinct function compared with *FT1*. We utilized a recently available sequenced diversity resource to reveal an allelic series for *FT-B3* (Cheng et al., 2024) and used this genetic diversity to investigate a possible role for lower-ambient-temperature signals. We observed that *FT-B3* expression responds to temperature and mediates the rate of inflorescence development. Finally, we observed that this temperature regulation is likely controlled through *miRNA319*, which is induced in the floral meristem and regulates a TCP transcription factor, *PROLIFERATING CELL FACTOR 5* (*PCF5*), that can associate with the promoter of *FT-B3*.

RESULTS

Allelic variation of *FT-B3* indicates a role in temperature adaptation

To understand and characterize the allelic diversity of *FT3* in bread wheat, we used recently generated genomic resources (Cheng et al., 2024). We obtained full genome sequences of *FT-A3*, *-B3*, and *-D3* for 1047 wheat cultivars, ranging from landraces collected in the 1930s through to elite modern wheat from across the world (Cheng et al., 2024). We began by characterizing the prevalence of a previously described deletion that includes *FT3* on the B sub-genome (Zikhali et al., 2017) and found that 22.6% of the cultivars contained a full-gene or full-gene and promoter deletion (Figure 1). By contrast, no full-gene deletion was identified in the A sub-genome, and only two cultivars showed a deletion of *FT3* in the D sub-genome. More broadly, very little allelic diversity was detected in *FT-A3* and *FT-D3* (Supplemental Figure 1), supporting previous characterization that the B sub-genome may be the critical genome in wheat for *FT3* function in environmental adaptation (Zikhali et al., 2017). Further analysis of the genomic collection identified 40 distinct *FT-B3* alleles, 24 of which are not observed in modern wheat

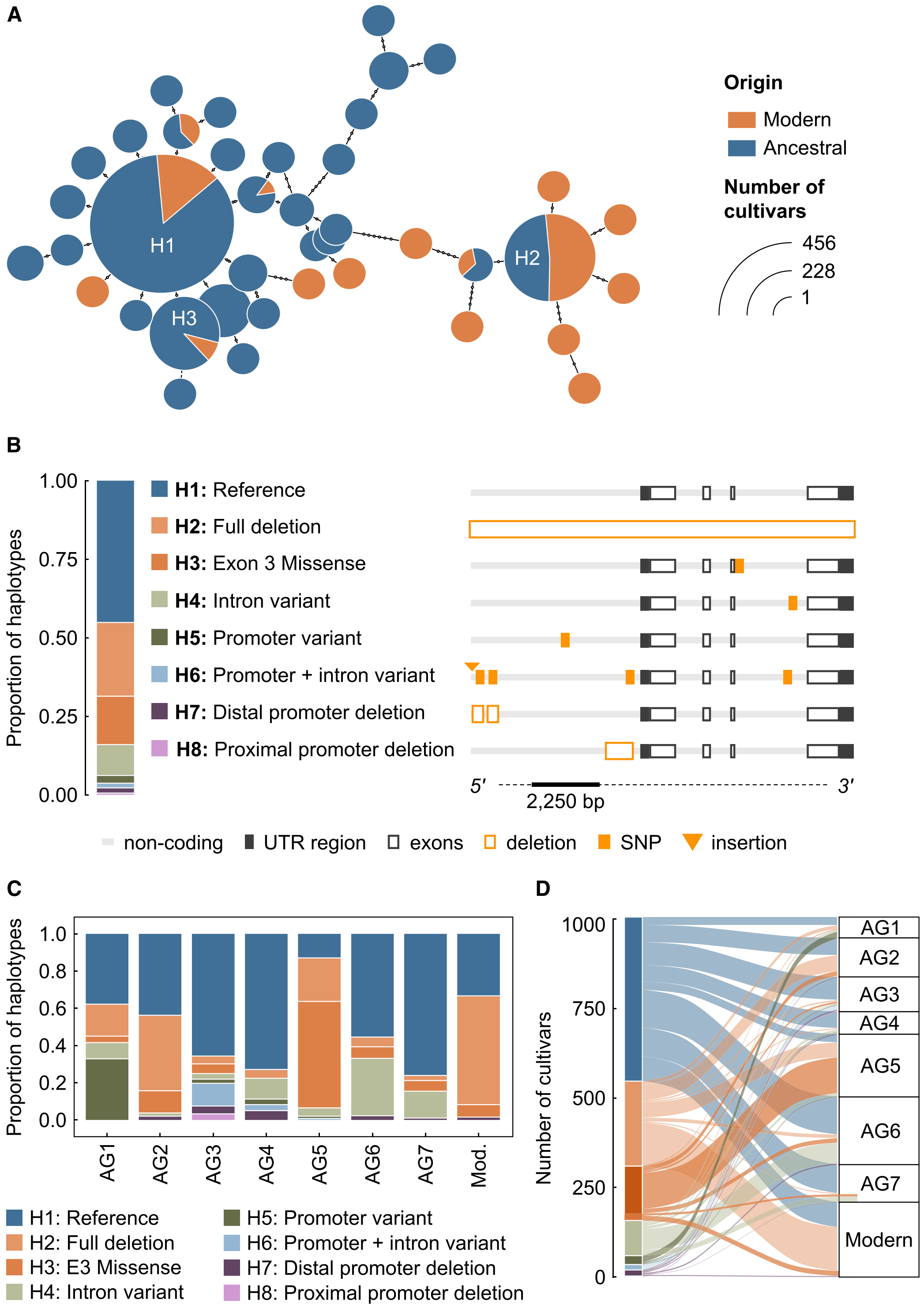

Figure 1. *FT-B3* alleles are associated with different ancestral groups.
(A) Haplotype network illustrating the differences between genomic *FT-B3* sequences of modern and ancestral cultivars. Small open circles indicate nucleotide changes between haplotypes.
(B) Stacked bar chart showing the proportions of *FT-B3* haplotypes across the examined cultivars (left) and gene models highlighting the positions of key SNPs and indels (right). Light gray represents non-coding DNA, including promoter regions; dark gray represents 3′ and 5′ UTRs; transparent black-edged boxes represent exons; and yellow/orange represents SNPs and indels.
(C) Stacked bar charts showing *FT-B3* haplotypes according to their representation in ancestral groups (AG) and modern wheat (Mod.).
(D) Alluvial plot representing the allelic diversity of *FT-B3* in AGs and modern cultivars.
E3, exon 3; UTR, untranslated region; SNP, single-nucleotide polymorphism; bp, base pair; H, haplotype; indel, insertion/deletion.

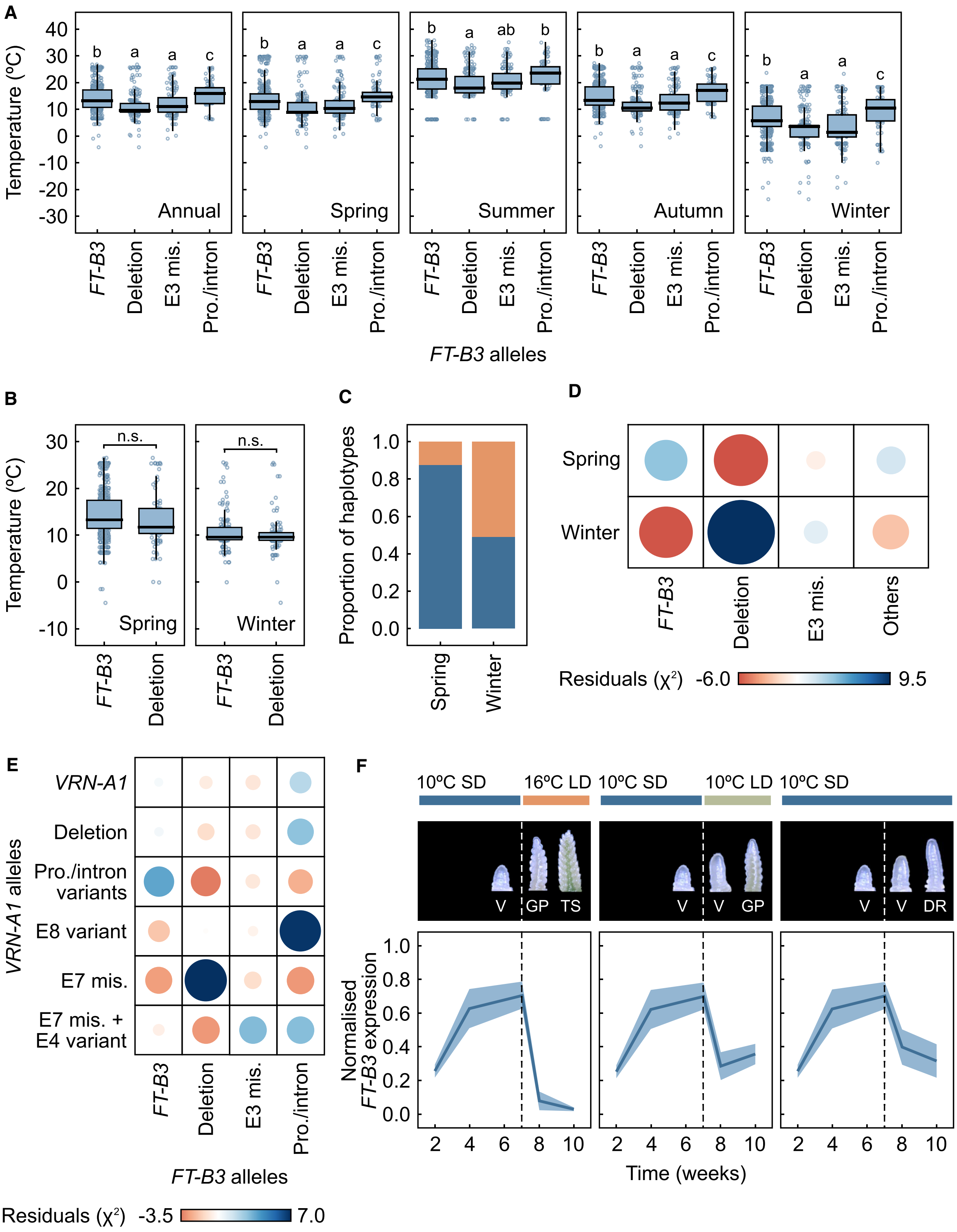

Figure 2. *FT-B3* alleles are associated with different environmental conditions.

(A) Boxplots showing the associations of major *FT-B3* haplotypes with average temperature. Each point represents an individual, distinct cultivar. Different letters indicate statistically significant differences among haplotypes *FT-B3* (H1), Deletion (H2), E3 mis. (H3), and Pro/intron (H4–8) (ANOVA, Tukey's HSD test, $p < 0.05$, $N = 98–458$).

(legend continued on next page)

(Figure 1A). Of these, only eight were found in four or more independent lines (Figure 1B), with the variation relative to the reference genome, Chinese Spring, listed in Supplemental Table 1. Using the ancestral group (AG) clustering (Cheng et al., 2024), distinct distributions of the alleles were observed (Figure 1C and 1D). The full-gene and promoter deletion (haplotype 2, H2) was enriched in modern cultivars but present in all AGs, which supports the idea of active selection of this allele but also makes determining the role of founder varieties much harder. In addition, we observed that certain alleles had been selected against in modern cultivars, including a missense mutation in the third exon (H3), which was most prevalent at 57% in AG5 (Figure 1C). For the major haplotypes, we have developed markers for their selection (Supplemental Figure 2).

As the allelic distribution of *FT-B3* in modern wheat suggested recent selection, we asked whether the allelic type was associated with the cultivation environment. To investigate this possibility, we used the recorded regional latitude and longitude for each cultivar (Cheng et al., 2024) and aligned it with publicly available temperature data (Berkeley Earth repository [2017–2019]). Each major haplotype group was then compared with yearly and seasonal temperature averages. This revealed that the *FT-B3* deletion tends to be associated with colder climates (Figure 2A). The same lower-temperature association was observed for an allele with a predicted missense mutation (Figure 2A, H3). Notably, H4, which contains a single-nucleotide polymorphism (SNP) in intron 3, showed a statistically significant association with warmer climates compared with H1, which is formed of the intact “wild-type” gene (Figure 2A). Given that the collection has full genome sequences and growth-habit assessments, we used this resource to further understand possible seasonal regulation via *FT-B3*. First, we tested the correlation between the *FT-B3* deletion and winter or spring growth habit (based on field trial analysis from spring-drilled field trials in Norwich, UK) (Figure 2B) (Cheng et al., 2024). There was co-inheritance of the full-gene deletion of *FT-B3* with winter growth habit cultivars (chi-squared test, $p < 0.05$), and 51% of winter-habit cultivars also contained a fully deleted *FT-B3* (Figure 2C and 2D). This strongly suggested that *FT-B3* alleles and growth habit, which is predominantly controlled through *VRN1*, may have been co-selected. We investigated the association between the allelic types of *FT-B3* and *VRN-A1* by chi-squared test. This showed that the spring allele of *VRN-A1* caused by a promoter deletion was associated with the reference or intact *FT-B3* allele, whereas *FT-B3* deletion and missense alleles were strongly correlated with *VRN-A1* alleles that were associated with the duration of vernalization, i.e., exon 4 and 7 SNPs, and therefore winter habit (Figure 2E) (Diaz et al., 2012).

On the basis of the co-inheritance, we hypothesized that the function of *FT-B3* may be linked to early plant growth. To investigate this possibility, *T. aestivum* cv. Cadenza plants were grown for 7 weeks at 10°C SD (8-h light:16-h dark), after which subsets were transferred to either 16°C LD (16-h light:8-h dark) or 10°C LD or maintained at 10°C SD for 3 weeks. Inflorescence development and gene expression were evaluated over a 10-week time course. Cadenza was used because it contains a full-length *FT-B3* allele in a spring-habit background. Inflorescence development was fastest under 16°C LD and slowest under 10°C SD (Figure 2F). This was supported by the expression of *FT-B1*, the LD florigen (Yan et al., 2006) (Supplemental Figure 3). As anticipated, *FT-B3* expression was highest under SD conditions (Figure 2F), although, interestingly, we also observed lower expression under warmer (16°C) compared with cooler (10°C) LD conditions.

FT-B3 expression is regulated by temperature and photoperiod

The haplotype analysis (Figure 1), combined with the developmental time course (Figure 2), suggested that *FT-B3* might have a broader role in relaying environmental signals, not just as an SD florigen. To test whether the reduction in gene expression observed between 10°C and 16°C LD was a result of slower apex development or an increase in temperature, we measured *FT-B3* expression under different temperatures and photoperiods. As genes involved in seasonal regulation are also commonly regulated by the circadian clock (Paajanen et al., 2021), we conducted four 24-h time courses in Cadenza. Under SD (8-h light:16-h dark), we observed diurnal regulation with a peak in expression just after dusk in both warmer (16°C) and cooler (10°C) conditions (Figure 3A). We compared this with expression under extended long days (ELDs; 22-h light:2-h dark) at the same temperatures and observed that *FT-B3* expression was extremely low at 16°C (Figure 3B). However, under the same ELD photoperiod, which would be anticipated to repress expression of an SD florigen, at 10°C, expression was still robustly observed to a peak level similar to that under SD (Figure 3B). This demonstrates that *FT-B3* expression is also induced by low temperature and that this regulation is enhanced by SD photoperiods, such that expression is still observed under warmer conditions. The expression analysis also identified diurnal regulation, in accordance with the existence of two MYB binding domains (AAATATC) within 2.5 kb of the *FT-B3* promoter (Supplemental Figure 4), which are known to be bound by circadian-related genes (Nagel et al., 2015).

We next asked how these signals might be relevant under field conditions, as lower temperatures and SDs are typical of winter

(B) Boxplots showing the association of the *FT-B3* deletion (H2) with temperature per spring/winter habit (Student’s *t*-test, $p < 0.05$, $N = 86–610$).
(C) Bar charts showing *FT-B3* haplotypes according to growth habit. Blue represents the reference allele (H1), and orange represents the *FT-B3* deletion (H2) from Figure 1.
(D) Heatmap depicting the correlations between the most representative alleles of *FT-B3* and growth habit. Circle area represents the absolute value of the Pearson residuals between the allele and the habit (chi-squared test of independence, $p < 0.05$), and color represents the correlation coefficient. Red and blue indicate negative and positive associations, respectively.
(E) Heatmap depicting the correlation between the most representative alleles of *FT-B3* and *VRN-A1*.
(F) Apex development (top) and *FT-B3* expression (bottom) over a 10-week time course under different environmental conditions. The photographs show representative apex developmental stages at weeks 6, 8, and 10. Lines represent mean fold change, and ribbons indicate SEM ($N = 3$). SD, short day (8-h light:16-h dark); LD, long day (16-h light:8-h dark); V, vegetative; DR, double ridge; GP, glume primordia; TS, terminal spikelet. E, exon; Pro, promoter; n.s., not significant.

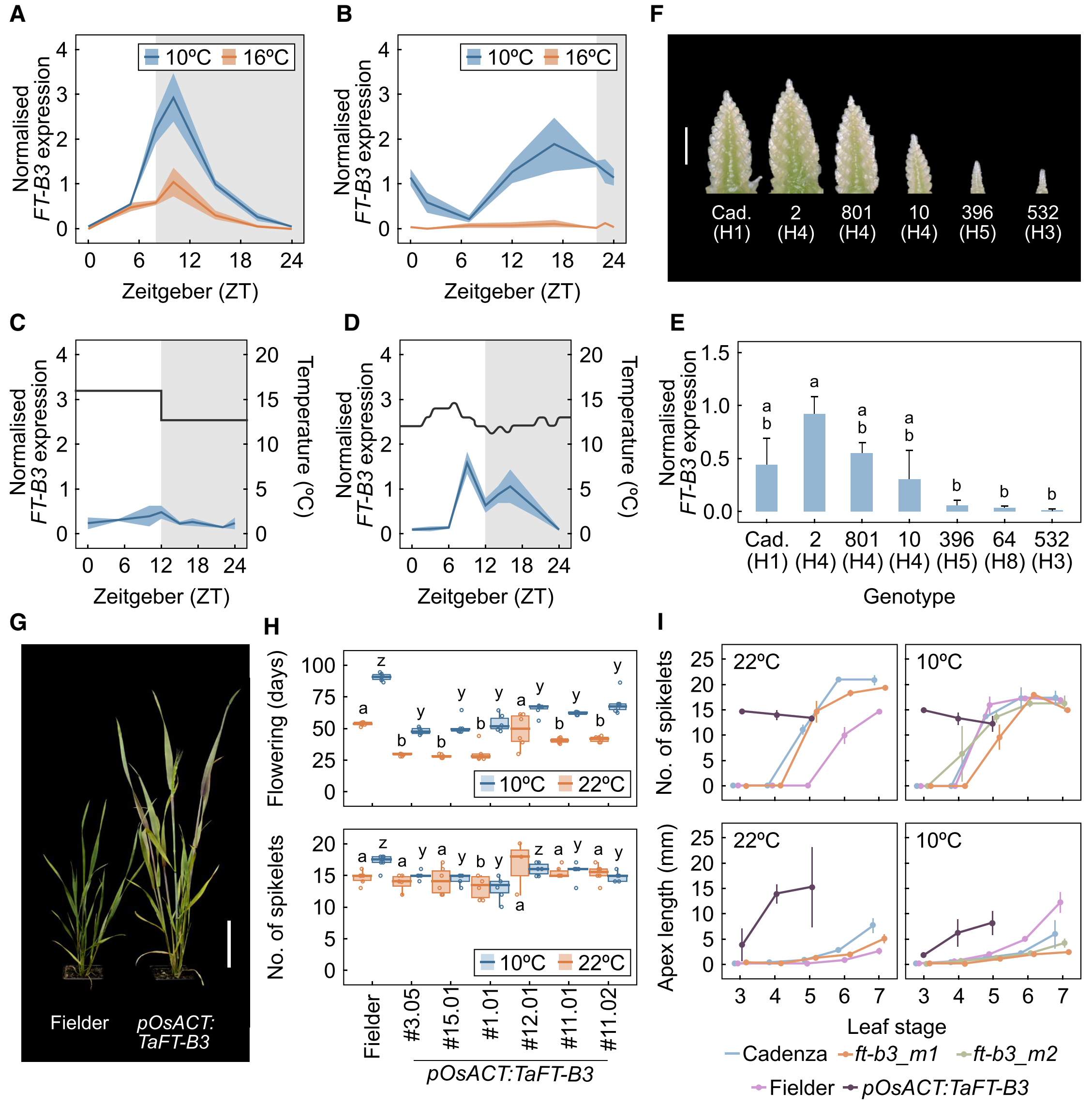

Figure 3. *FT-B3* is a temperature- and photoperiod-sensitive florigen.

(A and B) Ribbon plots showing the expression of *FT-B3* at 10°C (blue) and 16°C (orange) under **(A)** short-day (8-h light:16-h dark) and **(B)** extended long day (ELD; 22-h light:2-h dark) photoperiods.

(C and D) *FT-B3* expression under controlled **(C)** and environmentally fluctuating field temperature **(D)** conditions and a day-neutral (12-h light:12-h dark) photoperiod.

(E and F) *FT-B3* expression in leaf samples collected at day 22 of growth under ELD conditions **(E)** and representative images of the corresponding meristem developmental stage at day 45 **(F)**. Different letters indicate statistically significant differences among genotypes (ANOVA, Tukey's HSD test, $p < 0.05$, $N = 3$).

(G) Representative photographs of full plant development in the transgenic background cultivar (Fielder) and *pOsAct:FT-B3* plants at flowering; the scale bar indicates 9 cm.

(H) Boxplots showing flowering time (d.f.s., days from sowing) and number of spikelets in control Fielder and *pOsAct:FT-B3* plants at 10°C (blue) and 22°C (orange) under ELD conditions (22-h light:2-h dark). Different letters indicate statistically significant differences from Fielder within a temperature (ANOVA, Tukey's HSD test, $p < 0.05$, $N = 6$).

(I) Spikelet number and apex length for Cadenza and the Cadenza TILLING mutant BC_2 lines *ft-b3_m1* and *ft-b3_m2*, along with Fielder and *pOsAct:FT-B3* as used in **(H)** ($N = 3$).

Duration of lights off is indicated by a shaded box. H, haplotype.

conditions. We grew plants under a transition photoperiod of 12-h light:12-h dark and combined this with a warmer (16°C) light temperature and a cooler (10°C) dark temperature (Figure 3C). After 3 weeks under these conditions, *FT-B3* was barely expressed, suggesting that warm day temperatures repress *FT-B3* expression. However, under natural conditions, 16°C during day-neutral photoperiods is quite unusual. Using field-grown samples of the cv. Paragon, which is closely related to

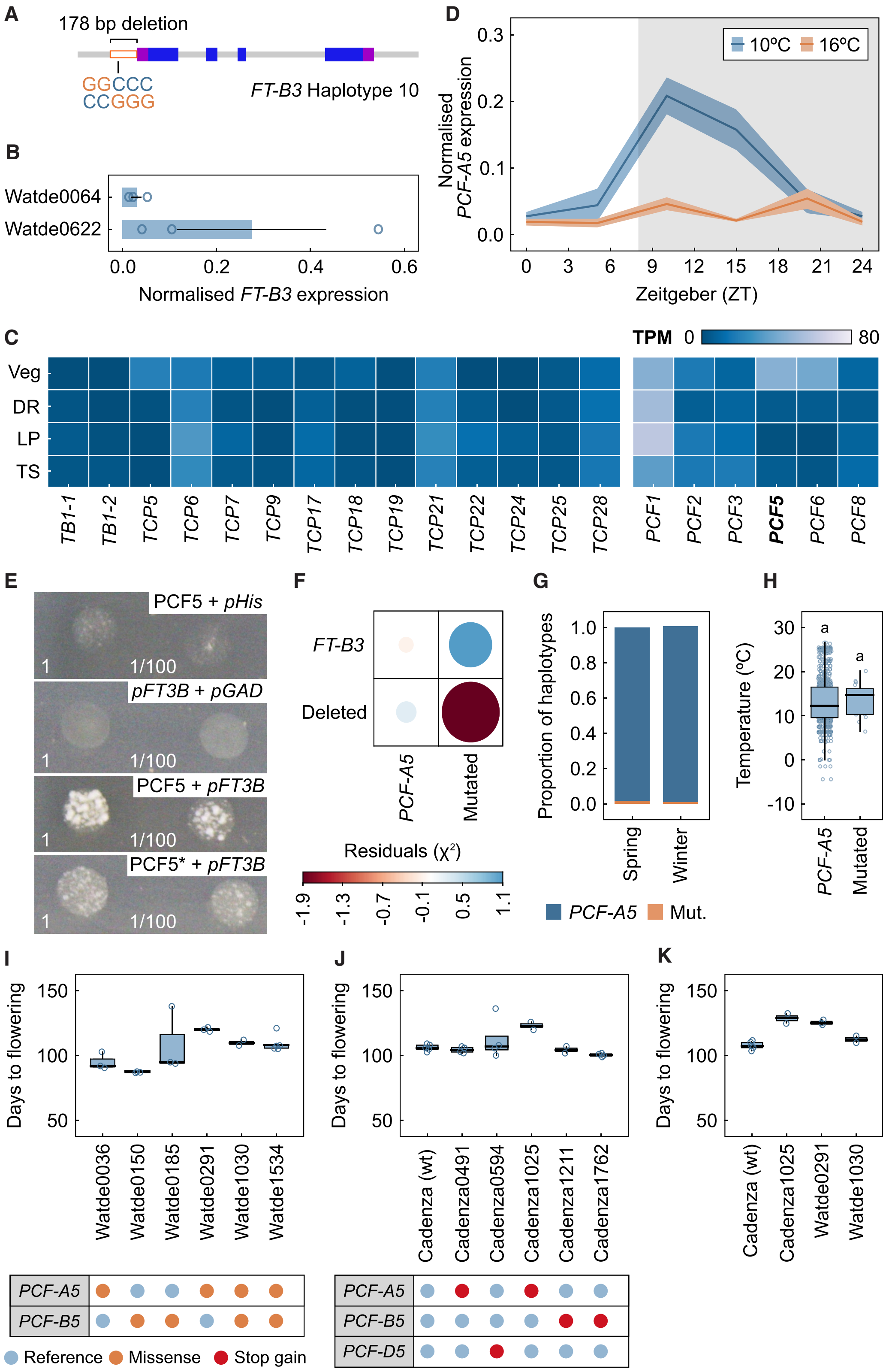

Figure 4. The TCP transcription factor *PCF5* associates with alleles of *FT-B3*.
(A and B) Schematic showing the position of the rare-allele promoter deletion in *FT-B3* **(A)** and *FT-B3* expression in two Watkins landraces that carry this deletion **(B)**.
(C) Heatmap of TCP transcription factor expression in cv. Paragon at different apex developmental stages.

(legend continued on next page)

Cadenza, we measured *FT-B3* expression across a 24-h time course when the natural photoperiod was 12-h light:12-h dark, resulting in a plant age of 160 days (Gauley and Boden, 2021). Here, *FT-B3* was robustly expressed under the variable temperatures experienced in the field, showing a pattern most similar to that observed under 10°C SD (Figure 3D).

Allelic variation in *FT-B3* alters the rate of apex development

Given the environmental sensitivity of *FT-B3*, we asked whether its allelic diversity was associated with any environmentally responsive developmental phenotypes. To test this possibility, we selected cultivars with different *FT-B3* alleles from our haplotype analysis (Supplemental Table 1). To remove the confounding effect of growth habit, we restricted our selection to cultivars with a reported spring habit (Cheng et al., 2024). The alleles included an intronic mutation (H4), a missense mutation (H3), a promoter deletion (H8), and a reference allele (H1), for which we selected Cadenza because it is the background for the hexaploid wheat TILLING resource (Krasileva et al., 2017) (for details of the alleles see Supplemental Table 4). Plants were grown at 10°C under ELD conditions and sampled after 3 weeks at zeitgeber (ZT) 17 for expression analysis. The developing apex of each plant was then dissected when Cadenza reached terminal spikelet, which occurred after 45 days of growth (Figure 3F). We observed that *FT-B3* expression corresponded to the inflorescence development rate of the analyzed cultivars, such that high *FT-B3* expression was associated with faster inflorescence development (Figure 3E and 3F). This was surprising, given the genetic diversity in the background of these cultivars, and strongly indicated that *FT-B3* is a major genetic regulator of early inflorescence development.

We therefore hypothesized that if we overexpressed *FT-B3*, the resulting plants would rapidly progress through apex development, even at lower temperatures. To test this possibility, we generated *pOsACT:FT-B3* lines in the readily transformed hexaploid wheat cv. Fielder (spring, photoperiod insensitive). To our surprise, these lines flowered exceptionally early compared with Fielder, with some individuals (n = 6 out of 32) flowering at the time of transplant from the transformation medium (Supplemental Figure 5A). We observed a range of flowering times in the independent transgenic events that produced the T_0 plants and were able to collect seed from the majority. We selected lines with a reliable increase in *FT-B3* expression (Supplemental Figure 5B) and grew F_1 plants under either 10°C or 22°C ELD conditions (Figure 3G, images from 22°C ELD). Flowering time was accelerated under both temperatures relative to Fielder (Figure 3H). In addition, spikelet number was consistently reduced relative to Fielder at 10°C (Figure 3H), and tiller number was reduced under both temperatures (Supplemental Figure 6). Ear length was also more affected at 10°C, whereas, surprisingly, plant height was relatively unaffected (Supplemental Figure 6).

To further characterize the allelic differences of *FT-B3* without the confounding factor of differences in genetic background, we identified two alleles in the hexaploid wheat TILLING population (Cadenza background [Krasileva et al., 2017]): Cadenza0790, a predicted deleterious (SIFT 0) missense variant (C>T, G114D), hereafter *ft-b3_m1*, and Cadenza0160, a splice-site mutation (G>A, intron 2), hereafter *ft-b3_m2*. We backcrossed these EMS-generated lines at least two times to reduce the influence of other mutations and selected homozygous lines for the respective *FT-B3* SNPs. To test the effect of these mutations on temperature-mediated floral development, we assessed flowering time and apex development at 10°C or 22°C ELD (Figure 3I and Supplemental Figure 7). Apex development was delayed in lines that lacked functional *FT-B3* alleles (Cadenza background) and significantly accelerated in *pOsACT:FT-B3* (Fielder background) (Figure 3I). Notably, the *pOsACT:FT-B3* lines flowered significantly earlier; however, the delayed apex development observed in the TILLING lines did not translate into a late-flowering phenotype (Supplemental Figure 7), once again suggesting that apex development and final flowering date can be controlled independently.

A temperature-sensitive TCP transcription factor can bind the promoter of *FT-B3*

To identify potential factors that control *FT-B3* expression, we searched for variations in the *FT-B3* promoter (Cheng et al., 2024). In addition to the fully deleted promoter, there was a rare allele that contained a 178-bp deletion in the proximal part of the *FT-B3* promoter, 160-bp upstream of the predicted start codon (Figure 4A; Supplemental Table 4). The allele was found only in four landrace cultivars, two from Afghanistan (WATDE0662 and WATDE0064), one from Iran (WATDE0836), and one of unknown origin (WATDE1040) (Cheng et al., 2024).

To gain an indication if this allele might be useful for dissecting the regulation of *FT-B3* expression, we measured gene expression in WATDE0662 and WATDE0064. Notably, both cultivars showed low expression of *FT-B3* under 10°C ELD, when we would usually expect *FT-B3* expression (Figure 4B). We analyzed transcription factor binding domains in the deleted region and identified a motif associated with type II TCP transcription factor binding (GGCCC). We used available RNA-sequencing data (Gauley et al., 2024) from the developing meristem to identify candidate TCP transcription factors expressed early in meristem development. *PCF5* was highly expressed during the vegetative stage (Figure 4C). This was of

(D) *PCF5* expression across the 24-h time course described in Figure 3A.

(E) Yeast-one-hybrid assay using PCF5 or truncated PCF5 with a premature stop codon (PCF5*) as the prey and the 178-bp *FT-B3* deleted promoter region as the bait.

(F) Heatmap of the association between *PCF-A5* and *FT-B3* alleles.

(G and H) Stacked bar chart of the distribution of *PCF-A5* alleles and growth habit **(G)** and the association of these lines with regional growth temperatures **(H)**.

(I–K) Days to flowering under 10°C extended long days (22-h light:2-h dark) for lines that vary in *PCF5* alleles from the Watkins (Watde) **(I)** and TILLING **(J)** collections and comparison of similar position mutations within these collections **(K)**.

Veg, vegetative; DR, double ridge; LP, lemma primordia; TS, terminal spikelet. Mut, mutant.

particular interest, as PCF5 has been linked to floral regulation and temperature responses in switchgrass (*Panicum virgatum*), rice, and *Arabidopsis* (Guo et al., 2013; Xie et al., 2017; Lan and Qin, 2020). In bread wheat, consistent with previous work (Zhao et al., 2018), we identified *PCF5*-like genes on the A, B, and D genomes. We examined the expression of *PCF5* under the same conditions as *FT-B3* (described in Figure 3A and 3B). The pattern under 10°C SD was strikingly similar to that of *FT-B3*, with a peak after dusk (Figure 4D compared with Figure 3A). *PCF5* expression was also responsive to temperature and was much lower under 16°C SD relative to 10°C SD (Figure 4D). The similarity between genes was lost under the longer photoperiod (Supplemental Figure 8). The co-expression of *FT-B3* and *PCF5* under SD may indicate that they function in the same pathway. To test whether PCF-B5 could associate with the 178-bp region from the *FT-B3* promoter, we performed yeast-one-hybrid assays using the promoter fragment as bait and PCF-B5 as prey, both sequences from Chinese Spring. These assays revealed an interaction between PCF-B5 protein and the *FT-B3* promoter fragment at 200 mM 3-AT (Figure 4E). We evaluated the specificity of this interaction by generating a TCP-domain mutant, C91*, for PCF-5 (PCF5*) and testing its binding with the same promoter fragment. Although PCF5* could still interact with the *FT-B3* fragment, it did so to a lesser degree (Figure 4E).

The similar temperature-responsive gene expression profiles and association of PCF-B5 with the *FT-B3* promoter region suggest that these genes could be in a common temperature-sensitive pathway. We therefore sought to determine how such an interaction could regulate *FT-B3* expression and whether PCF5 had a role in wheat floral development. First, we tested whether natural alleles of *PCF5* showed environmental or growth habit selection similar to that observed for *FT-B3* alleles. To identify allelic variation in *PCF5*, we used the genetic diversity set (Cheng et al., 2024). In this collection, the D sub-genome contained no variation, and the A and B sub-genomes had reduced levels of variation compared with *FT3*. On the A sub-genome, we identified seven variants of *PCF-5* and eight of *PCF-B5* (Supplemental Table 5; Supplemental Figure 9). Given the small number of individual cultivars with variants, we considered these as a single mutated class for association analysis with the *FT-B3* alleles. Interestingly, we observed that the *FT-B3* deletion and mutated *PCF-A5* never occurred together, suggesting a possible epistatic relationship between *FT-B3* and *PCF-A5* alleles, although such a pattern could also have occurred purely by chance (Figure 4F). The *PCF-A5* and *B5* alleles showed no association with growth habit (Figure 4F), with association occurring only when considered in combination with the previously described *FT-B3* distribution (Supplemental Figure 10). In addition, when we associated the *PCF5* alleles against growing temperature, we also did not observe any temperature interaction (Figure 4G). To directly investigate the role of PCF-5 in wheat floral development, we used the few allelic types in *PCF5* that were predicted via missense mutations to produce fewer functional PCF5 proteins in either the A or B sub-genome or both (Supplemental Table 5) but retained a functional *FT-B3*. These six cultivars were grown under 10°C ELD; although these conditions were not those under which we would expect *PCF5* to have the largest phenotypic effect based on the gene expression data (Supplemental Figure 8), hexaploid wheat grown under 10°C SD rarely completes its development. We aimed to minimize flowering diversity from known vernalization and photoperiod genes, but the small number of available *PCF5* alleles made this extremely difficult. We could control for a spring background, but minimizing the *Ppd-1* diversity constricted the available panel to a few lines (Supplemental Table 5; Figure 4I). In general, we observed a trend toward later flowering for lower-function *PCF-A5* alleles (cf. WATDE0291, 1030, 1534, and 0036); however, no conclusions could be drawn. To address the variable backgrounds, we identified *PCF5* alleles from the Cadenza TILLING collection (Krasileva et al., 2017) that contained predicted loss-of-function mutations for *PCF5* (Supplemental Table 4; Figure 4J). Interestingly, one of the TILLING lines contained a stop codon in *PCF-A5* (Cadenza1025, Gln283*), in a location similar to that of a natural mutation in WATDE1030 and WATDE0291 (Figure 4K). This analysis showed that under 10°C LD, mutations in *PCF-A5* resulted in delayed flowering (Figure 4K), which was lost when plants were grown under 22°C ELD (Supplemental Figure 11).

A temperature-responsive pre-miR319 is expressed during floral development

Several TCP transcription factors are regulated through highly conserved microRNA (miRNA) pathways (Palatnik et al., 2003; Kubota et al., 2017; Fang et al., 2021). Of particular interest with regard to the lower-ambient-temperature regulation of *PCF5*, and therefore *FT-B3*, was the regulation of *PCF5 by miR319/JAW* in rice, with a possible link to frost tolerance (Yang et al., 2013). We therefore aimed to determine whether a similar temperature-linked regulation might exist in wheat. Through sequence analysis of *PCF-5* across a diverse selection of grass species, we identified a highly conserved miR319 binding site (Figure 5A). The same miR319 binding sequence identified in rice was conserved in hexaploid bread wheat on the A and B sub-genomes (Figure 5A). This suggested that a pre-miRNA319 sequence should be present in the wheat genome and, through comparison with the rice pre-miR319, we were able to identify a putative 193-bp pre-miR319 ortholog in wheat, annotated as *ENSRNA050023569-T1*, a *pre-miRNA156* (Ensembl Plants v.59), on chromosome 3A—hereafter, *pre-miR319* (Figure 5B). We tested for expression of *pre-miR319* and were unable to detect any *pre-miR319* expression in leaf tissue under any temperature examined (24-h time course at 10°C or 16°C, leaf tissue before and after the reproductive transition). This result was consistent with previous observations in rice (Yang et al., 2013). We did, however, detect expression in the meristem at the vegetative and double-ridge stages, and this expression was significantly higher at 22°C than at 10°C. The localization of *pre-miR319* expression was similar to that of *PCF5*, as *PCF5* expression can be measured in leaf tissue but is higher in vegetative and floral meristems (Figure 5C–5E). When we analyzed the transcript distribution in apex samples from a time series of field-grown plants (Gauley et al., 2024), we observed that the level of *PCF5* transcript decreased as temperatures naturally warmed, especially across the miR319 binding site (Supplemental Figure 12).

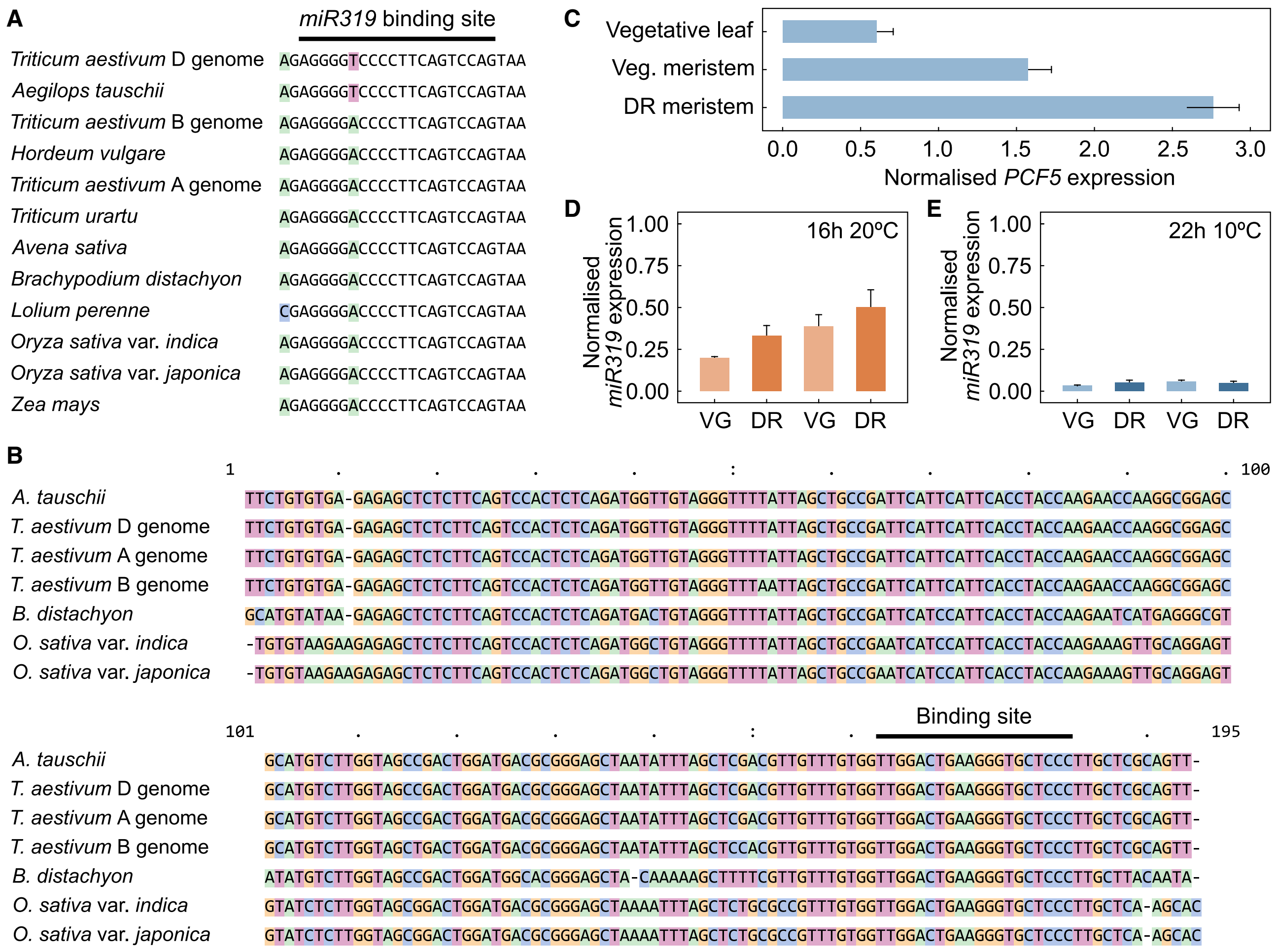

Figure 5. Identification of conserved *pre-miR319* and binding sites in hexaploid wheat.

(A) Alignment of the miR319 binding regions from *PCF5* genes in multiple grass species, with SNPs highlighted in color.

(B) Alignment of putative *pre-miR319*, based on the rice (*O. sativa*) sequence as a guide, with the miR319 sequence highlighted by the binding site bar.

(C) *PCF5* expression in meristem and leaf tissue.

(D and E) *pre-miR319* expression under 20°C with a 16-h light:8-h dark photoperiod assessed using two primer sets **(D)** and under 10°C with a 22-h light:2-h dark photoperiod assessed using the same primer sets **(E)**.

VG, vegetative apex; DR, double ridge apex. N = 3; error bars represent as SE.

DISCUSSION

Selection during cultivation can enable the identification of candidate genes for targeted adaptation

Hexaploid wheat has been under cultivation for thousands of years, enabling its adaptation to different environments and fortuitously providing an exceptional genetic resource for understanding environmental adaptation. This genetic resource is now meeting the genomics revolution, providing an unprecedented level of sequenced material (Cheng et al., 2024). Using this resource, we identified a significant association between the deletion of *FT-B3* and winter growth habit in modern cultivars (Figure 1A). Through association with average seasonal growth temperatures at the reported variety locations, we observed that varieties containing the deletion tended to be grown in areas with lower average autumn and winter temperatures (Figure 2A), suggesting that *FT-B3* has been selected against in areas with colder winters, consistent with similar reports in barley (Casao et al., 2011).

The combined allelic assessment of *FT-B3* with the *VRN-A1* alleles suggests that FT-B3 may have an important role in development (Figure 2E). Through transgenic overexpression of *FT-B3*, we found that *FT-B3* can be as potent a floral activator as *FT-B1* (Li et al., 2015) (Figure 3H and Supplemental Figure 5). Given that *FT-B3* was not detected in the vernalization-related QTL studies that identified *FT-B1*, but that there is genetic variation between winter and spring habits at the *FT-B3* loci, the regulation and timing of *FT-B3* expression may be key to its function in wheat.

FT genes monitor different signals from the environment in bread wheat

We observed that *FT-B3* expression responds to both SDs and lower ambient temperatures (Figure 3A) and that this response has an effect early in apex transition and development (Figure 3F). This suggests that the regulation of *FT-B3* expression and its localization are important in regulating wheat floral

development and potentially more broadly in coordinating the action of the FT genes. The early developmental impact of FT3 under SDs and low temperatures places its activity before that of FT2 (Shaw et al., 2019) but potentially after that of FT1 (Yan et al., 2006; Gauley and Boden, 2021). Thus, rather than acting as a cascade of gatekeepers at each meristem stage in cereals, FTs instead appear to act in response to developmental stage and environmental cues, enabling the coordination of plant development across an entire growing season (Gauley and Boden, 2021).

Expression data for *FT-B3* under SD conditions (Figure 3A) and in the field (Figure 3D) suggest that *FT-B3* expression is strongly gated at the start of the day, between ZT0 and ZT5, during which time *FT-B3* is less transcriptionally responsive to environmental conditions such as temperature. These data also suggest that *FT-B3* has a role in integrating both temperature and photoperiod signals under field conditions. This highlights that *FT-B3* activity accelerates early floral development in a temperature-dependent fashion, with *FT-B3* being induced at lower temperatures. Furthermore, it strongly suggests that the regulation of early floral development can be separated from that of whole-plant morphology, as accelerated flowering is normally associated with shorter plant height owing to the reduction in development time.

FT-B3 can be regulated by a TCP transcription factor

Because overexpression of *FT-B3* led to such significant acceleration of floral development (Figure 3I), it was clear that regulation and localization of this gene were crucial to its usual function *in planta*. Notably, in *Arabidopsis*, rice, and wheat BRC1, TEOSINTE BRANCHED 1 (TB1 respectively regulates FT and FT1, respectively (Dixon et al., 2018). TB1 belongs to a large family of TCP transcription factors, a number of which have been linked to seasonal regulation of floral development in other plant species

(Cubas et al., 1999; van Es et al., 2019). Interestingly, we could identify TCP binding sites in the promoter of *FT-B3*, specifically in a rare allele of *FT-B3* where 178 bp of the promoter was deleted. The TCP binding sites in this allele most closely aligned with those bound by the type II TCP factors, which include PCF5, a TCP factor that has been linked to developmental regulation (Zhao et al., 2018). We confirmed that PCF5 could bind the 178-bp region through yeast-one-hybrid assays (Figure 4E). This does not mean that PCF5 is the only transcriptional regulator of *FT-B3*, nor does it indicate in which direction this regulation occurs. However, it clearly suggests a route through which *FT-B3* transcription could be partially regulated. This possibility was supported by the highly similar expression profiles of *FT-B3* and *PCF5* under SD photoperiods (Figures 3A and 4D).

Interestingly, when we considered temperature-responsive regulation by PCF5, it was clear that adaptive selection had actually focused on *FT-B3*. The *PCF-5* genes showed very little allelic variation (Figure 4G). This may be because *PCF5* is involved in a much wider range of signaling functions, as observed in rice and switchgrass, and selection on *PCF5* would therefore have resulted in greater changes than those observed through *FT-B3*.

Temperature sensing and response pathways can be conserved in plants despite species-specific adaptations

The identification of PCF5 as a regulator of *FT-B3* expression was particularly interesting, as *PCF5* orthologs in rice are regulated by a miRNA (Yang et al., 2013; Lan and Qin, 2020). The miRNA (*JAW*) is temperature sensitive in a manner opposite to that of *PCF5* and *FT3*. Inspection of the *PCF5* sequence revealed a highly conserved *miR319* binding site (Figure 5A), and using the rice pre-mRNA sequence as a guide, we identified a candidate *pre-miR319* in the wheat genome (Figure 5B). Expression analysis of this transcript showed that its expression was higher under warmer temperatures (Figure 5D). Although additional genetic evidence is required to confirm a role for this miRNA within the pathway, our findings strongly indicate that wheat contains a conserved temperature-sensing pathway that, rather than regulating leaf morphology, has been recruited to regulate early-stage floral development (Figure 5F).

Using the *FT-B3* allelic series for adaptation in multiple environments

The results presented here suggest that *FT-B3* is an excellent candidate to further adapt our cereal crops in response to SDs and low temperature to regulate early-stage floral development. Regulation of this stage is important, as it determines multiple yield-potential traits, including spikelet number, in wheat. In addition, controlling this early-stage and seasonal response for floral development may offer a route to enable rapid development and, therefore, combined with early-flowering alleles, a method to ensure that a crop flowers before damaging summer heat and drought.

This suggests that *FT-B3* is an excellent candidate for environmental adaptation. For example, selection of the deleted allele could slow growth towards the end of winter and thus encourage an increase in spikelet number while avoiding spring frosts. Alternatively, selection of *FT-B3* could enable the development of shorter-season winter and spring cultivars, which are becoming increasingly important with changing climate patterns (Figure 6). To enable such selection, we have developed markers for the major alleles identified here (Supplemental Figure 2) and have identified cultivars that contain these variations. Going forward, it will be important to further understand how the *FT-B3* alleles interact with different genetic backgrounds, particularly with regard to the photoperiod pathway regulated by *PPD-1*.

METHODS

Germplasm

This study used genotypes of spring and winter hexaploid wheat (*T. aestivum*) from the Watkins wheat collection (Cheng et al., 2024). Backcrossed germplasm was derived from Cadenza TILLING mutants from (Krasileva et al., 2017; Rakszegi et al., 2010) as detailed in Supplemental Table 4. Transgenic *pOsAct:FT-B3* lines were developed with the JIC wheat transformation and gene editing facility (John Innes Centre, Norwich, UK). The construct contains the *FT-B3* (*TraesCS1B02G351100*) coding region as determined from Ensembl Plants (https://plants.ensembl.org/index.html).

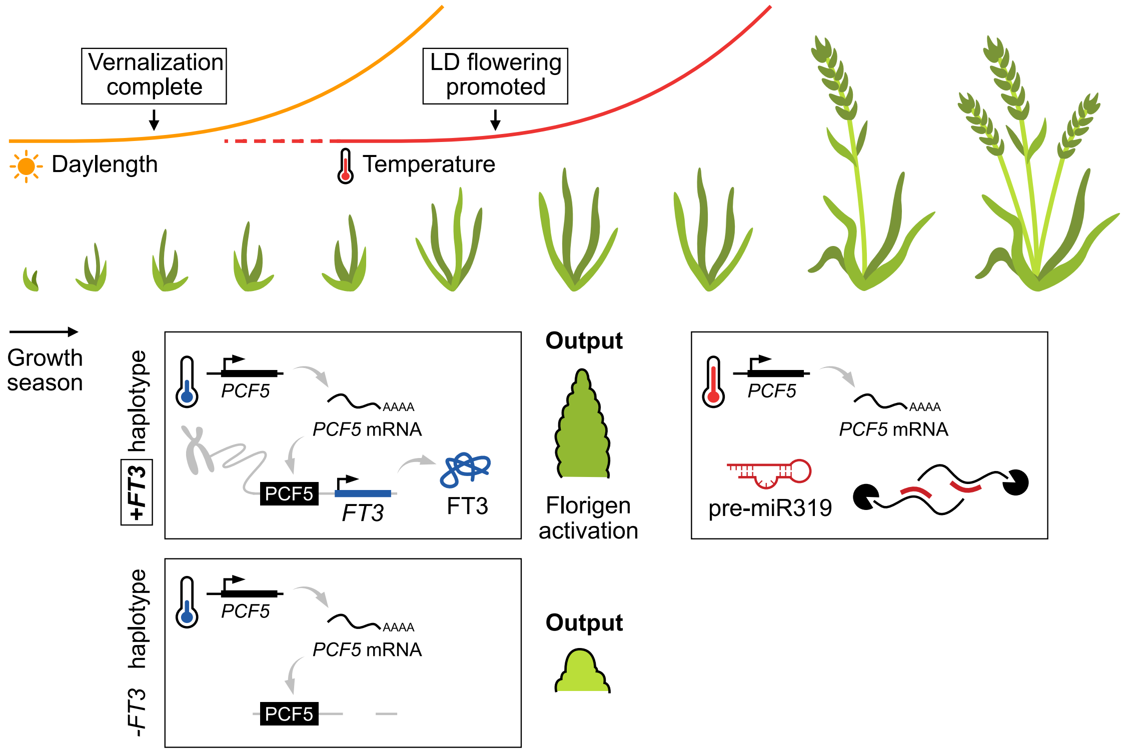

Figure 6. Proposed summary pathway for the role of a short-day, low-temperature florigen in wheat development.
The schematic shows increasing day length (yellow line) and temperature (red line), with the two major environmentally regulated flowering triggers of vernalization and long-day (LD) photoperiods highlighted relative to the visual plant developmental stage. There is a developmental gap between the completion of vernalization and the promotion of LD flowering, which is where FT3 acts. The molecular mechanism relating to temperature regulation is shown in the boxes for the two major *FT3* haplotypes. Under lower temperatures (blue), *PCF5* is expressed and translated into protein, which enables increased expression of *FT3* and accelerates early floral meristem development. Under higher temperatures, *PCF5* transcript is targeted for miR319-mediated degradation; *miR319* is expressed more under warmer temperatures. In the haplotype lacking *FT3*, floral apex development is slowed across the environmental duration that matches with short days and lower temperatures.

Haplotype analysis

Gridded monthly land data for average temperature between 2017 and 2019 were extracted from http://berkeleyearth.org/data/. The latitude and longitude coordinates provided for the collection were matched to the gridded data, which were obtained over a 1 × 1 degree grid and therefore cover a larger area than the single longitude/latitude coordinates. Lines that lacked longitude and latitude coordinates were excluded from the analysis. Genomic variation data for the bioinformatic analysis were downloaded from https://wwwg2b.com/ (Cheng et al., 2024) and used the AG and haplotype analysis generated in Cheng et al. (2024) using HAPPE v.0.1.4. Subsequent analysis was performed using R statistical software (Zhang et al., 2023). The geneHapR package was used to extract gene haplotypes from the genomic data following the developers' guidelines (RStudio Team, 2020). The table2hap and hap_summary functions were used to read the genomic data and obtain summarized variant information. The haplotype network in Figure 1A was obtained using the get_hapNet function with default settings. Haplotypes present in fewer than four accessions were excluded from further analysis, and data visualization was performed using tidyverse packages.

Promoter domain analysis

Sequences were obtained from Ensembl Plants release 59 and analyzed using PlantPan (Chow et al., 2016).

Genotyping and cloning

Samples containing ~2 cm of the youngest leaf were collected, frozen in liquid nitrogen, and ground using a TissueLyser (Qiagen, Hilden, Germany). DNA was extracted using cotton lysis buffer and resuspended in 50 μl of TE buffer. Genotyping was performed using PACE genotyping in 96-well plates as described in Dixon et al. (2018) with primers described in Supplemental Table 2. The 178-bp region of the *FTB-3* promoter, *PCF-5*, and *PCF-5b* carrying the 91C* mutation were synthesized by Azenta. All sequences were identified from Ensembl plants release 59.

Growing conditions

The expression studies across 24 h used Cadenza grown in controlled growth cabinets (Sanyo) under 10°C or 16°C SD (8-h light/16-h dark), ELD (22-h light/2-h dark), or day neutral (12-h light/12-h dark) conditions with full available light intensity. Leaf samples were collected in increments throughout the diel growth period for gene expression analysis. Field-based expression analyses were performed on wild-type cv. Paragon plants grown in 1-m^2 plots at field sites of the John Innes Centre based at Church Farm, John Innes Centre, Bawburgh, Norfolk, UK (52°62′25.7″N, 1°21′83.2″E). These field-grown plants experienced natural increases in photoperiod and temperature as described previously (Gauley and Boden, 2021). The T_0 *pOsAct:FT-B3* lines were grown in a controlled environment room under a 16-h light/8-h dark photoperiod at 20°C. Subsequent phenotyping experiments were performed at either 10°C or 22°C (22-h light/2-h dark). For Watkins *FT-B3* expression analysis, plants were grown at 10°C (22-h light/2-h dark). Images of inflorescence meristems were obtained at the time of sampling at ZT17. Experiments involving expression of *miRNA319* in leaves, inflorescences, and phloem were performed on Cadenza plants grown in controlled glasshouses maintained at 20°C (16-h light/ 8-h dark). Inflorescence expression was

compared with that of Cadenza plants grown in controlled growth cabinets (Sanyo) maintained at 10°C (22-h light/2-h dark).

Tissue sampling

Plants were sampled at 3 weeks of age, unless otherwise stated. For image analysis, meristem images were obtained after 45 days of growth, when Cadenza had reached the terminal spikelet stage. For leaf tissue, the most recently emerged leaf was sampled, placed in a 2-ml collection tube, and immediately frozen in liquid nitrogen. Three biological replicates were collected for all experiments. For inflorescence meristem phenotyping and gene expression analysis, representative inflorescences were examined using a Keyence binocular dissecting microscope and imaged using a Keyence color camera. At least three inflorescences per genotype were phenotyped and imaged at each time point.

RNA isolation and expression analysis

RNA was extracted from leaf tissue using the Spectrum Plant Total RNA Kit (Sigma-Aldrich, St. Louis, MO, USA) and from developing inflorescences using the RNeasy Plant Mini Kit (Qiagen). RNA was treated with RQ1 DNase I (Promega, Madison, WI, USA) and then reverse transcribed with SuperScript III reverse transcriptase (Life Technologies, Carlsbad, CA, USA) according to the manufacturer's instructions. Quantitative RT–PCR was performed on a Bio-Rad CFX Connect Real-Time System using GoTaq qPCR and RT–qPCR Systems (Promega) according to the manufacturer's instructions. The oligonucleotides used for RT–qPCR are listed in Supplemental Table 3. Candidate gene expression in leaf and inflorescence tissue was normalized using *TraesCS6D02G145100* as described in Dixon et al. (2018). All RT–qPCR data points are the average of three biological replicates, with two technical replicates performed for each reaction.

Yeast-one-hybrid system

The prey construct was generated using a template from a plasmid containing *PCF-B5* with SmaI and SacI restriction sites on either end, synthesized by GeneWiz (South Plainfield, NJ, USA). Using restriction digest cloning (NEB, Ipswich, MA, USA), *PCF-B5* was transferred to *pGAD-T7*, transformed into competent *Escherichia coli* cells (NEB), and confirmed by Sanger sequencing (Azenta, https://www.azenta.com/). The bait construct was generated by synthesizing the *FT-B3* promoter fragment into pHIS2 by Azenta. The *pGAD-T7:PCFB5* was transformed into *Saccharomyces cerevisiae* strain Y187 following the manufacturer's protocol (MPBio, Irvine, CA, USA), and successful transformants were selected by growth on plates without leucine. *FT-B3* promoter sequences in *pHIS2* were transformed into yeast in the same manner and plated onto agar plates without tryptophan. *FT-B3* was then transformed using the protocol above but with *PCF-B5* yeast as the host, and yeast were plated onto agar plates without leucine or tryptophan. To test the interaction, cells were streaked on agar without leucine, tryptophan, or histidine. The strength of the interaction was tested using the inhibitor 3-AT at 2.5, 5, and 10 mM concentrations. Controls included transformation of the empty plasmid.

Statistical tests and sample sizes

Statistical tests are described in the text and/or figure legends and were performed using base functions of R statistical software (RStudio Team, 2020). In brief, data from each individual experiment were first tested for normality by graphical inspection and a Shapiro–Wilk test. Statistical comparisons between conditions or genotypes for normally distributed data were performed with Student's *t*-test (2 groups) or ANOVA followed by Tukey's HSD test (>2 groups). For data that significantly deviated from a normal distribution, non-parametric tests were used instead: Wilcoxon tests (2 groups) or Kruskal–Wallis tests followed by Wilcoxon signed-rank tests with a Benjamini and Hochberg correction (>2 groups). Statistically significant associations between categorical variables were evaluated with a chi-squared test of independence. For gene expression analyses, three biological replicates from independent plants were used. For other experiments, sample sizes are indicated in the figure legends and consisted of at least three independent plants.

FUNDING

This research was supported through funding to L.D. via a UKRI FLF MR/S031677/1, Rank Prize Funds New Lecturer Award, and start-up funds from the University of Leeds. A Molecules to Landscapes grant from BBSRC supported H.T. S.G. was supported by BBSRC ISP "BBSRC Strategic Programme in Designing Future Wheat (DFW)" (BB/P016855/1); L.D. and S.G. were supported by BBSRC ISP "BBSRC Institute Strategic Programme: Delivering Sustainable Wheat (DSW)" (BB/X011003/1). S.C. was supported by the the National Key Research and Development Program of China (2023YFF1000100 and 2023YFA0914600).

ACKNOWLEDGMENTS

We thank Dr. Ann Kirsten-Koehler for extracting the temperature data from the Berkeley repository. The EMS-mutagenized population of bread wheat *cv.* Cadenza was developed and characterised by Dr. Andy Phillips at Rothamsted (UK) and Dr. Cristobal Uauy at John Innes Centre (UK). No conflict of interest is declared.

AUTHOR CONTRIBUTIONS

A.G., I.L., P.G.-S., H.T., D.H., and L.D. conceived, designed, and performed the experiments and analyses. L.D., P.G.-S., and A.G. wrote the initial manuscript, and all authors confirmed its accuracy. P.G.-S. produced final data visualizations and scientific drawings. W.H., S.H., and M.S. generated the transgenic FT3-OX. S.G. and S.C. performed analysis and shared the Watseq data prior to publication.

SUPPLEMENTAL INFORMATION

Supplemental information is available at *Plant Communications Online*.

Received: March 4, 2025
Revised: April 21, 2025
Accepted: September 1, 2025
Published: September 5, 2025

REFERENCES

Abe, M., Kobayashi, Y., Yamamoto, S., Daimon, Y., Yamaguchi, A., Ikeda, Y., Ichinoki, H., Notaguchi, M., Goto, K., and Araki, T. (2005). FD, a bZIP Protein Mediating Signals from the Floral Pathway Integrator FT at the Shoot Apex. Science **309**:1052–1056.

Aguilar-Martinez, J.A., Poza-Carrion, C., and Cubas, P. (2007). Arabidopsis BRANCHED1 acts as an integrator of branching signals within axillary buds. Plant Cell **19**:458–472.

Bennett, T., and Dixon, L.E. (2021). Asymmetric expansions of FT and TFL1 lineages characterize differential evolution of the EuPEBP family in the major angiosperm lineages. BMC Biol. **19**:181.

Casao, M.C., Igartua, E., Karsai, I., Lasa, J.M., Gracia, M.P., and Casas, A.M. (2011). Expression analysis of vernalization and day-length response genes in barley (Hordeum vulgare L.) indicates that VRNH2 is a repressor of PPDH2 (HvFT3) under long days. J. Exp. Bot. **62**:1939–1949.

Chen, Q., Payyavula, R.S., Chen, L., Zhang, J., Zhang, C., and Turgeon, R. (2018). FLOWERING LOCUS T mRNA is synthesized in specialized companion cells in Arabidopsis and Maryland Mammoth tobacco leaf veins. Proc. Natl. Acad. Sci. USA **115**:2830–2835.

Cheng, S., Feng, C., Wingen, L.U., Cheng, H., Riche, A.B., Jiang, M., Leverington-Waite, M., Huang, Z., Collier, S., Orford, S., et al. (2024). Harnessing landrace diversity empowers wheat breeding. Nature **632**:823–831.

Chow, C.N., Zheng, H.Q., Wu, N.Y., Chien, C.H., Huang, H.D., Lee, T.Y., Chiang-Hsieh, Y.F., Hou, P.F., Yang, T.Y., and Chang, W.C. (2016). PlantPAN 2.0: an update of plant promoter analysis navigator

for reconstructing transcriptional regulatory networks in plants. Nucleic Acids Res. **44**:D1154–D1160.

Cubas, P., Lauter, N., Doebley, J., and Coen, E. (1999). The TCP domain: a motif found in proteins regulating plant growth and development. Plant J. **18**:215–222.

Diaz, A., Zikhali, M., Turner, A.S., Isaac, P., and Laurie, D.A. (2012). Copy number variation affecting the Photoperiod-B1 and Vernalization-A1 genes is associated with altered flowering time in wheat (Triticum aestivum). PLoS One **7**:e33234.

Dixon, L.E., Greenwood, J.R., Bencivenga, S., Zhang, P., Cockram, J., Mellers, G., Ramm, K., Cavanagh, C., Swain, S.M., and Boden, S.A. (2018). TEOSINTE BRANCHED1 Regulates Inflorescence Architecture and Development in Bread Wheat (Triticum aestivum). Plant Cell **30**:563–581.

Dreisigacker, S., Burgueno, J., Pacheco, A., Molero, G., Sukumaran, S., Rivera-Amado, C., Reynolds, M., and Griffiths, S. (2021). Effect of Flowering Time-Related Genes on Biomass, Harvest Index, and Grain Yield in CIMMYT Elite Spring Bread Wheat. Biology **10**:855.

Fang, Y., Zheng, Y., Lu, W., Li, J., Duan, Y., Zhang, S., and Wang, Y. (2021). Roles of miR319-regulated TCPs in plant development and response to abiotic stress. The Crop Journal **9**:17–28.

Faure, S., Higgins, J., Turner, A., and Laurie, D.A. (2007). The FLOWERING LOCUS T-like gene family in barley (Hordeum vulgare). Genetics **176**:599–609.

Gauley, A., and Boden, S.A. (2021). Stepwise increases in FT1 expression regulate seasonal progression of flowering in wheat (Triticum aestivum). New Phytol. **229**:1163–1176.

Gauley, A., Pasquariello, M., Yoshikawa, G.V., Alabdullah, A.K., Hayta, S., Smedley, M.A., Dixon, L.E., and Boden, S.A. (2024). Photoperiod-1 regulates the wheat inflorescence transcriptome to influence spikelet architecture and flowering time. Curr. Biol. **34**:2330–2343.

Guo, S., Xu, Y., Liu, H., Mao, Z., Zhang, C., Ma, Y., Zhang, Q., Meng, Z., and Chong, K. (2013). The interaction between OsMADS57 and OsTB1 modulates rice tillering via DWARF14. Nat. Commun. **4**:1566.

Halliwell, J., Borrill, P., Gordon, A., Kowalczyk, R., Pagano, M.L., Saccomanno, B., Bentley, A.R., Uauy, C., and Cockram, J. (2016). Systematic Investigation of FLOWERING LOCUS T-Like Poaceae Gene Families Identifies the Short-Day Expressed Flowering Pathway Gene, TaFT3 in Wheat (Triticum aestivum L.). Front. Plant Sci. **7**:857.

Jaeger, K.E., and Wigge, P.A. (2007). FT protein acts as a long-range signal in Arabidopsis. Curr. Biol. **17**:1050–1054.

Kikuchi, R., Kawahigashi, H., Ando, T., Tonooka, T., and Handa, H. (2009). Molecular and functional characterization of PEBP genes in barley reveal the diversification of their roles in flowering. Plant Physiol. **149**:1341–1353.

Krasileva, K.V., Vasquez-Gross, H.A., Howell, T., Bailey, P., Paraiso, F., Clissold, L., Simmonds, J., Ramirez-Gonzalez, R.H., Wang, X., Borrill, P., et al. (2017). Uncovering hidden variation in polyploid wheat. Proc. Natl. Acad. Sci. USA **114**:E913–E921.

Kubota, A., Ito, S., Shim, J.S., Johnson, R.S., Song, Y.H., Breton, G., Goralogia, G.S., Kwon, M.S., Laboy Cintrón, D., Koyama, T., et al. (2017). TCP4-dependent induction of CONSTANS transcription requires GIGANTEA in photoperiodic flowering in Arabidopsis. PLoS Genet. **13**:e1006856.

Lan, J., and Qin, G. (2020). The Regulation of CIN-like TCP Transcription Factors. Int. J. Mol. Sci. **21**:4498.

Laurie, D.A., Pratchett, N., Snape, J.W., and Bezant, J.H. (1995). RFLP mapping of five major genes and eight quantitative trait loci controlling flowering time in a winter × spring barley (Hordeum vulgare L.) cross. Genome **38**:575–585.

Li, C., Lin, H., and Dubcovsky, J. (2015). Factorial combinations of protein interactions generate a multiplicity of florigen activation complexes in wheat and barley. Plant J. **84**:70–82.

Lv, B., Nitcher, R., Han, X., Wang, S., Ni, F., Li, K., Pearce, S., Wu, J., Dubcovsky, J., and Fu, D. (2014). Characterization of FLOWERING LOCUS T1 (FT1) gene in Brachypodium and wheat. PLoS One **9**: e94171.

Mulki, M.A., Bi, X., and von Korff, M. (2018). FLOWERING LOCUS T3 Controls Spikelet Initiation But Not Floral Development. Plant Physiol. **178**:1170–1186.

Nagel, D.H., Doherty, C.J., Pruneda-Paz, J.L., Schmitz, R.J., Ecker, J.R., and Kay, S.A. (2015). Genome-wide identification of CCA1 targets uncovers an expanded clock network in Arabidopsis. Proc. Natl. Acad. Sci. USA **112**:E4802–E4810.

Niwa, M., Daimon, Y., Kurotani, K.i., Higo, A., Pruneda-Paz, J.L., Breton, G., Mitsuda, N., Kay, S.A., Ohme-Takagi, M., Endo, M., et al. (2013). BRANCHED1 interacts with FLOWERING LOCUS T to repress the floral transition of the axillary meristems in Arabidopsis. Plant Cell **25**:1228–1242.

Paajanen, P., Lane de Barros Dantas, L., and Dodd, A.N. (2021). Layers of crosstalk between circadian regulation and environmental signalling in plants. Curr. Biol. **31**:R399–R413.

Palatnik, J.F., Allen, E., Wu, X., Schommer, C., Schwab, R., Carrington, J.C., and Weigel, D. (2003). Control of leaf morphogenesis by microRNAs. Nature **425**:257–263.

Pieper, R., Tomé, F., Pankin, A., and von Korff, M. (2021). FLOWERING LOCUS T4 delays flowering and decreases floret fertility in barley. J. Exp. Bot. **72**:107–121.

Shaw, L.M., Lyu, B., Turner, R., Li, C., Chen, F., Han, X., Fu, D., and Dubcovsky, J. (2019). FLOWERING LOCUS T2 regulates spike development and fertility in temperate cereals. J. Exp. Bot. **70**:193–204.

Tamaki, S., Matsuo, S., Wong, H.L., Yokoi, S., and Shimamoto, K. (2007). Hd3a protein is a mobile flowering signal in rice. Science **316**:1033–1036.

Taoka, K.i., Ohki, I., Tsuji, H., Furuita, K., Hayashi, K., Yanase, T., Yamaguchi, M., Nakashima, C., Purwestri, Y.A., Tamaki, S., et al. (2011). 14-3-3 proteins act as intracellular receptors for rice Hd3a florigen. Nature **476**:332–335.

Rakszegi, M., Kisgyorgy, B.N., Tearall, K., Shewry, P.R., Láng, L., Phillips, A., Bedő, Z., et al. (2010). Diversity of agronomic and morphological traits in a mutant population of bread wheat studied in the Healthgrain program. Euphytica **174**:409–421.

RStudio Team. (2020). RStudio: Integrated Development for R. RStudio (PBC).

van Es, S.W., van der Auweraert, E.B., Silveira, S.R., Angenent, G.C., van Dijk, A.D.J., and Immink, R.G.H. (2019). Comprehensive phenotyping reveals interactions and functions of Arabidopsis thaliana TCP genes in yield determination. Plant J. **99**:316–328.

Wigge, P.A., Kim, M.C., Jaeger, K.E., Busch, W., Schmid, M., Lohmann, J.U., and WeigeL, D. (2005). Integration of Spatial and Temporal Information During Floral Induction in Arabidopsis. Science **309**:1056–1059.

Worland, A.J., Borner, A., Korzun, V., Li, W.M., Petrovíc, S., and Sayers, E.J. (1998). The influence of photoperiod genes on the adaptability of European winter wheats. Euphytica **100**:385–394.

Xie, Q., Liu, X., Zhang, Y., Tang, J., Yin, D., Fan, B., Zhu, L., Han, L., Song, G., and Li, D. (2017). Identification and Characterization of microRNA319a and Its Putative Target Gene, PvPCF5, in the Bioenergy Grass Switchgrass (Panicum virgatum). Front. Plant Sci. **8**:396.

Yamamoto, T., Kuboki, Y., Lin, S.Y., Sasaki, T., and Yano, M. (1998). Fine mapping of quantitative trait loci Hd1, Hd2 and Hd3, controlling heading date of rices as single Mendelian factors. Theor. Appl. Genet. **97**:37–44.

Yan, L., Fu, D., Li, C., Blechl, A., Tranquilli, G., Bonafede, M., Sanchez, A., Valarik, M., Yasuda, S., and Dubcovsky, J. (2006). The wheat and barley vernalization gene VRN3 is an orthologue of FT. Proc. Natl. Acad. Sci. USA **103**:19581–19586.

Yan, L., Loukoianov, A., Tranquilli, G., Helguera, M., Fahima, T., and Dubcovsky, J. (2003). Positional cloning of the wheat vernalization gene VRN1. Proc. Natl. Acad. Sci. USA **100**:6263–6268.

Yan, L., Loukoianov, A., Blechl, A., Tranquilli, G., Ramakrishna, W., San Miguel, P., Bennetzen, J.L., Echenique, V., and Dubcovsky, J. (2004). The Wheat VRN2 Gene Is a Flowering Repressor Down-Regulated by Vernalization. Science **303**:1640–1644.

Yang, C., Li, D., Mao, D., Liu, X., Ji, C., Li, X., Zhao, X., Cheng, Z., Chen, C., and Zhu, L. (2013). Overexpression of microRNA319 impacts leaf morphogenesis and leads to enhanced cold tolerance in rice (Oryza sativa L.). Plant Cell Environ. **36**:2207–2218.

Zhang, R., Jia, G., and Diao, X. (2023). geneHapR: an R package for gene haplotypic statistics and visualization. BMC Bioinf. **24**:199.

Zhao, J., Zhai, Z., Li, Y., Geng, S., Song, G., Guan, J., Jia, M., Wang, F., Sun, G., Feng, N., et al. (2018). Genome-Wide Identification and Expression Profiling of the TCP Family Genes in Spike and Grain Development of Wheat (Triticum aestivum L.). Front. Plant Sci. **9**:1282.

Zikhali, M., Wingen, L.U., Leverington-Waite, M., Specel, S., and Griffiths, S. (2017). The identification of new candidate genes Triticum aestivum FLOWERING LOCUS T3-B1 (TaFT3-B1) and TARGET OF EAT1 (TaTOE1-B1) controlling the short-day photoperiod response in bread wheat. Plant Cell Environ. **40**:2678–2690.

Plant Communications, Volume *6*

Supplemental information

Fine-tuning wheat development for the winter to spring transition

Adam Gauley, India Lacey, Pablo González-Suárez, Harry Taylor, Dominique Hirsz, Sadiye Hayta, Mark Smedley, Wendy Harwood, Simon Griffiths, Shifeng Cheng, and Laura Dixon

SUPPLEMENTARY INFORMATION

Fine tuning wheat development for the winter to spring transition.

Adam Gauley[1, 5], India Lacey[1], Pablo González-Suárez[1,6], Harry Taylor[1,7], Dominique Hirsz[1,4], Sadiye Hayta[2], Mark Smedley[2], Wendy Harwood[2], Simon Griffiths[2], Shifeng Cheng[3, 8], Laura Dixon[1,4]*

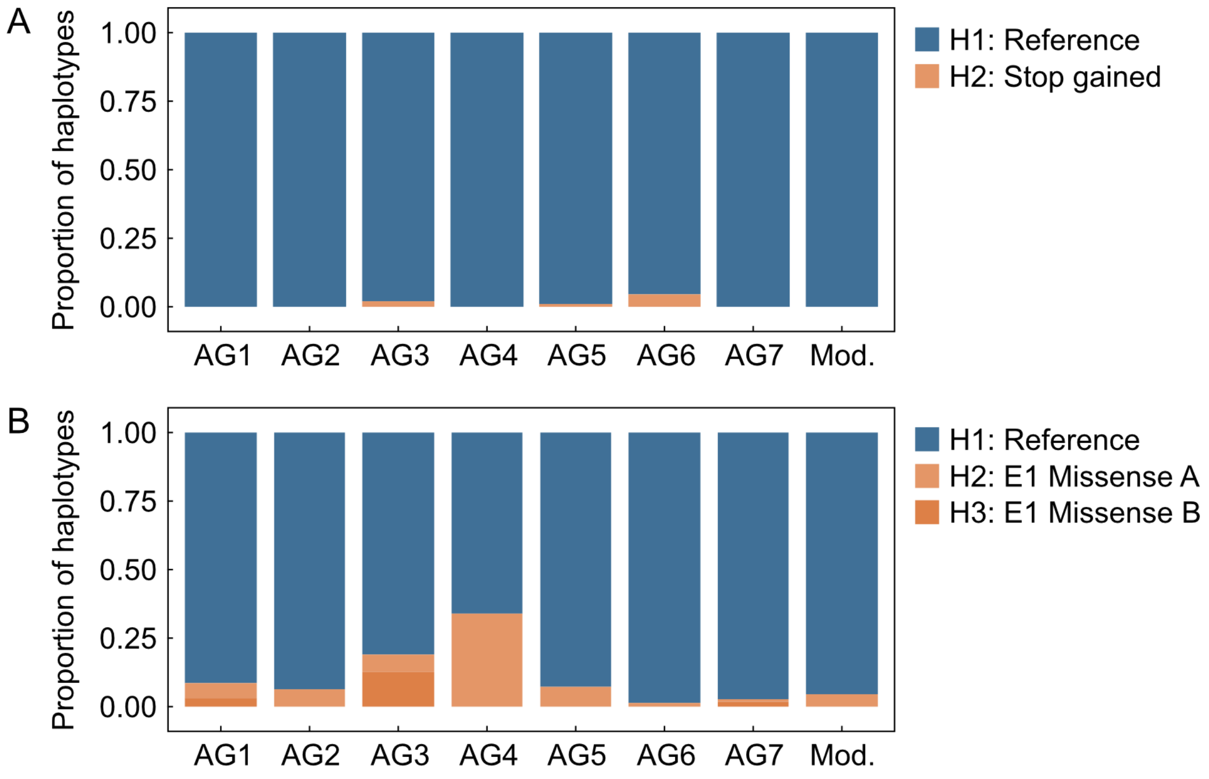

Supplementary Figure S1. Allelic diversity of *FT3* is reduced in the A and D subgenomes

Bar charts showing *FT-A3* haplotypes according to representation in ancestral groups (AG) and modern wheat (Mod.) (**A**). Bar charts showing *FT-D3* haplotypes according to representation in ancestral groups (AG) and modern wheat (Mod.) (**B**). E: exon

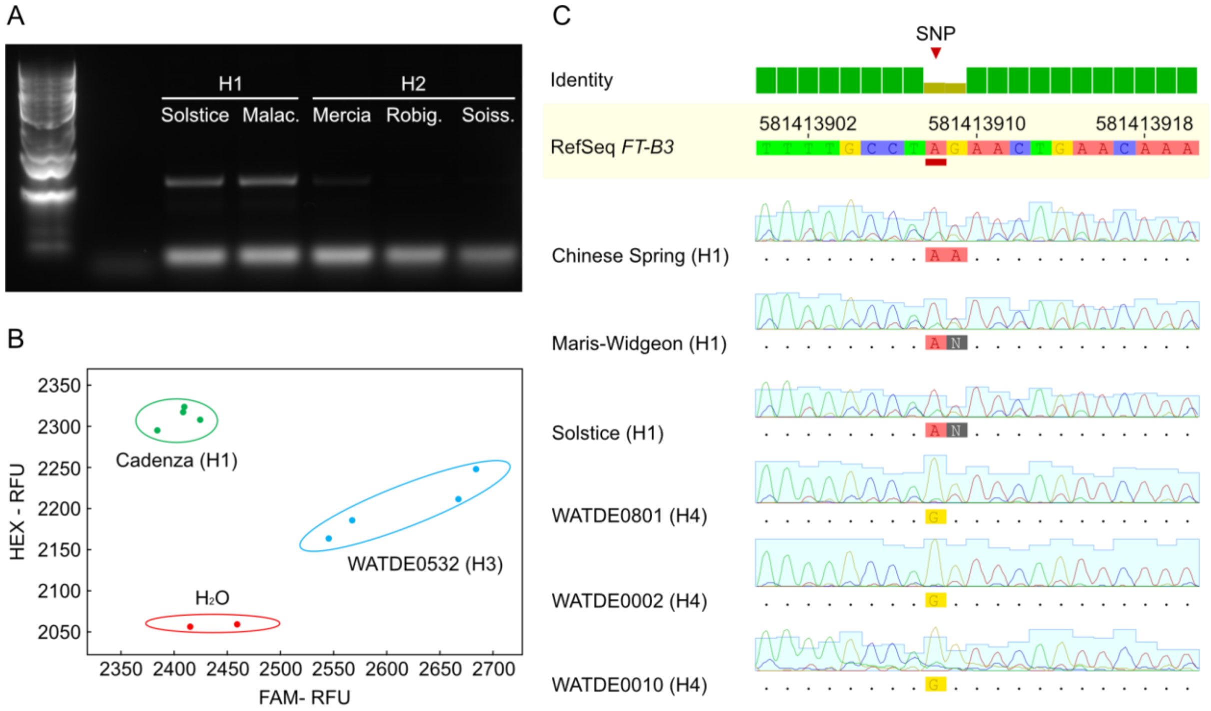

Supplementary Figure S2. Example outputs of markers developed to identify the major *FT-B3* haplotypes

Example 1% agarose gel highlighting expected band for non-deletion versus deleted (H2) *FT-B3* (**A**). Example KASP genotyping output for distinguishing haplotype H3 (**B**). Example sequencing reads for identification of haplotype H4 SNP (**C**). All using primers detailed in **Supplementary Table 2**. H: haplotype

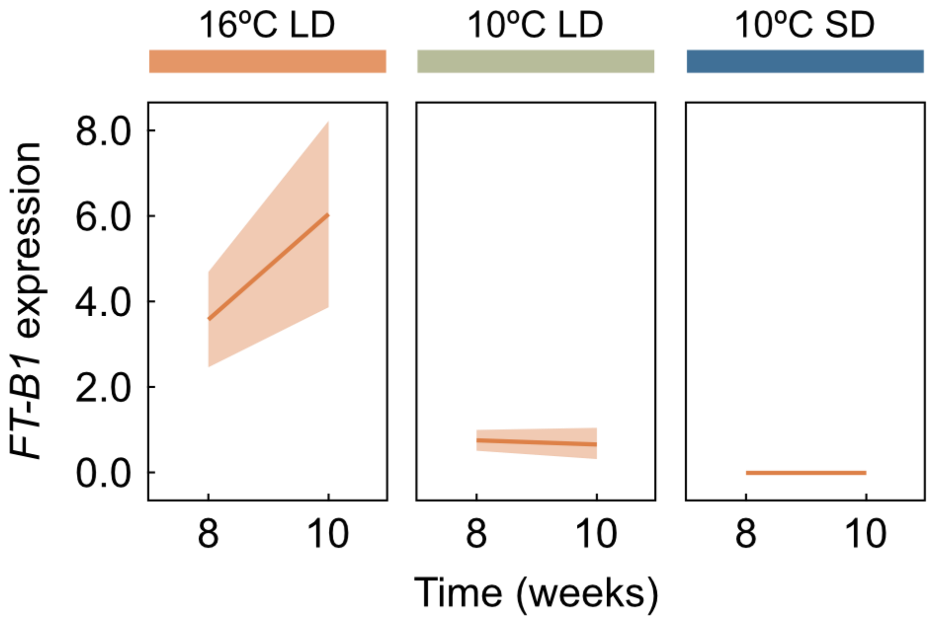

Supplementary Figure S3. *FT-B1* expression across 10-week developmental time-course under varying conditions. As described in **Figure 2F** for *FT-B3* expression (N=3). Lines indicate the average of biological replicates per time point, ribbons indicate standard error of the mean.

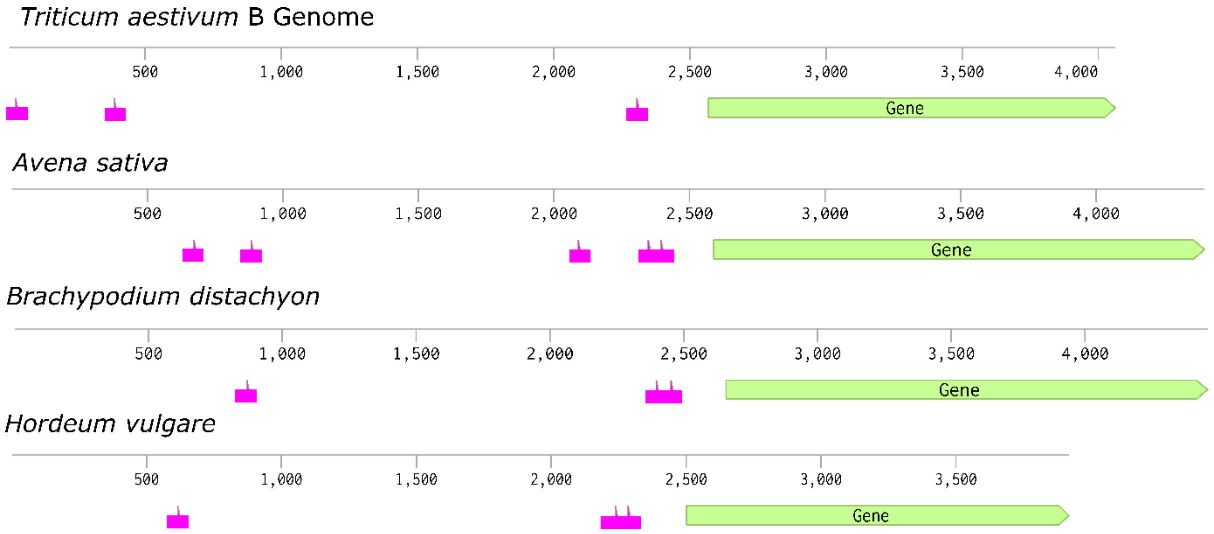

Supplementary Figure S4. MYB binding sites in the *FT-B3* promoter region

FT-B3 gene and promoter map for *Triticum aestivum* and close gene orthologs in *Avena sativa*, *Brachypodium distachyon* and *Hordeum vulgare*. MYB binding domains (AAATATC) within the 2.5KB upstream promoter region are indicated by a magenta box. Gene mapping is indicated in green.

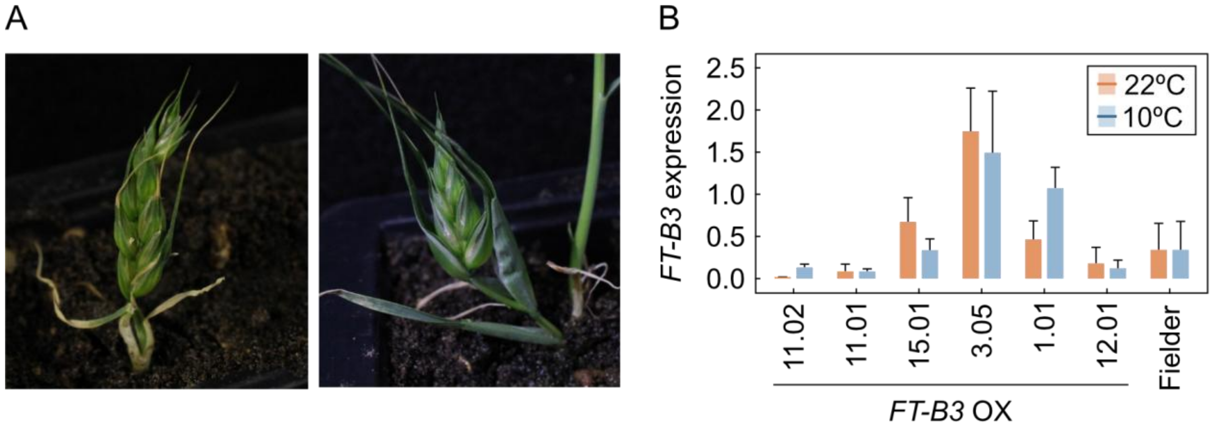

Supplementary Figure S5. Characterization of *FT-B3* overexpression lines

Two example photographs of the exceptionally early flowering phenotype observed in some of the T_0 FT-B3 overexpressing transgenic lines (*pOsAct:FT-B3)* (*cv.* Fielder background) (**A**). Expression of *FT-B3* at 10°C and 22°C in different transgenic lines (N=3) (**B**). Height of bars indicate the average of biological replicates per combination of line and temperature, error bars indicate standard error.

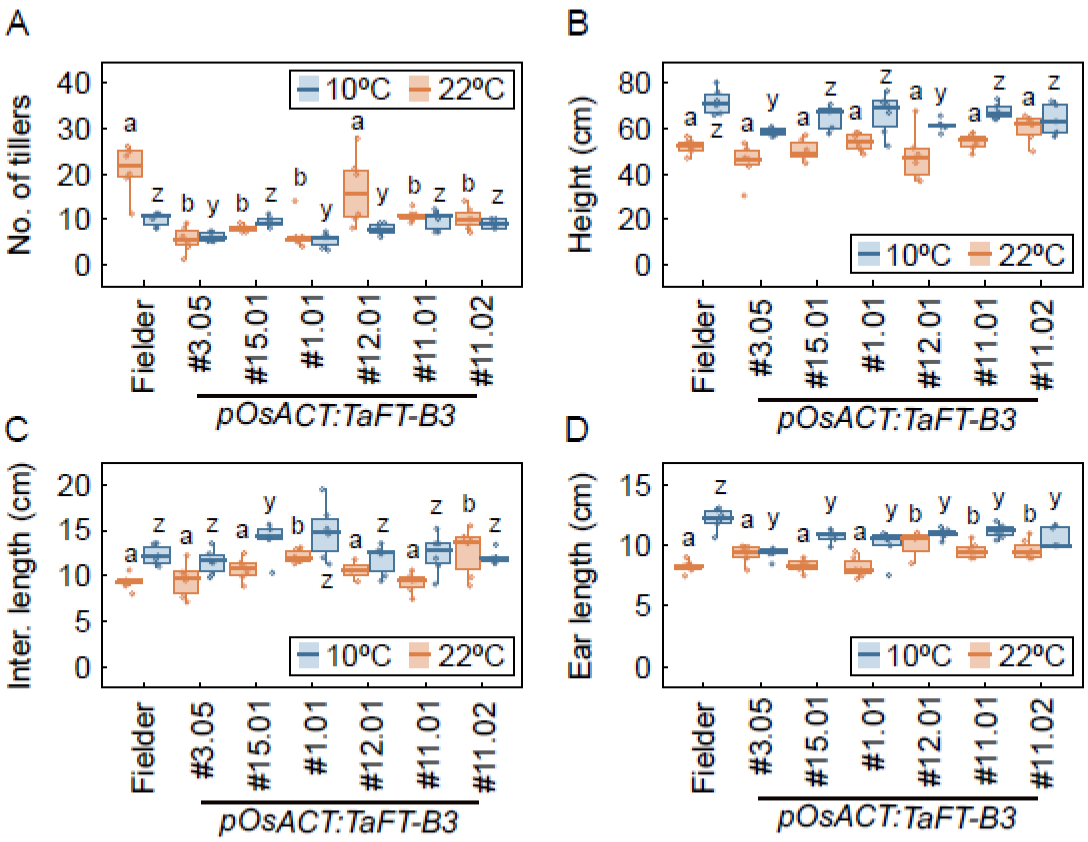

Supplementary Figure S6. Trait characterization of *FT-B3* overexpression lines

Final stage developmental trait measurement for *FT-B3* overexpressing lines (*pOsAct:FT-B3*) at 10°C (blue) and 22°C (orange) ELD (22 h light: 2 h dark). Number of fertile tillers (**A**), plant height (**B**), average final internode (peduncle) length (**C**) and ear length of the first spike (**D**). Different letters indicate statistical differences to *cv.* Fielder within a temperature (ANOVA, Tukey HSD test, $p<0.05$, N=6).

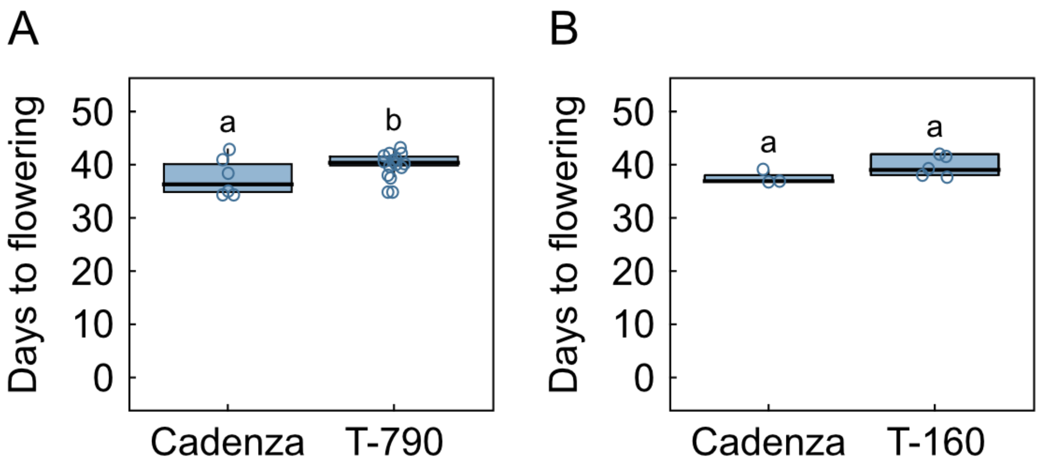

Supplementary Figure S7. Days to flowering of TILLING mutant lines for *FT-B3*

Days to flowering from BC_2F_2 homozygous lines from original TILLING line Cadenza0790 (T-790; *ft-b3*_m1), where different letters indicate statistical differences between lines (Student's t test, $p<0.05$, N=6-14) (**A**) and BC_1F_2 for Cadenza0160 (T-160; *ft-b3*_m2) where different letters indicate statistical differences between lines (Wilcoxon test, $p<0.05$, N=3-5) (**B**) with Cadenza as the background control.

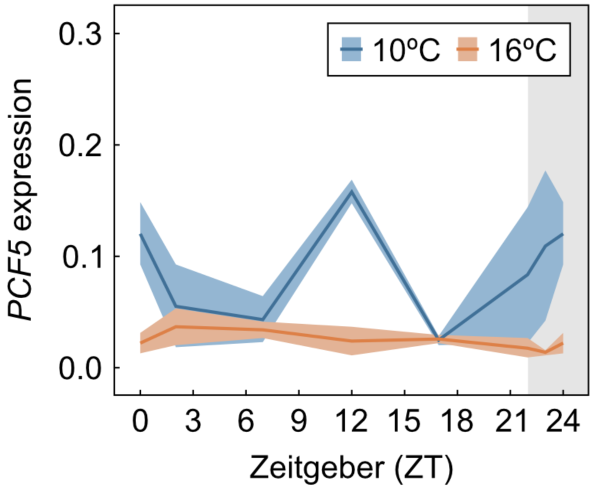

Supplementary Figure S8. Expression of *PCF5* in ELD.

As described in **Figure 3B** for *FT-B3* (N=3). Lines indicate the average of biological replicates per time point, ribbons indicate standard error.

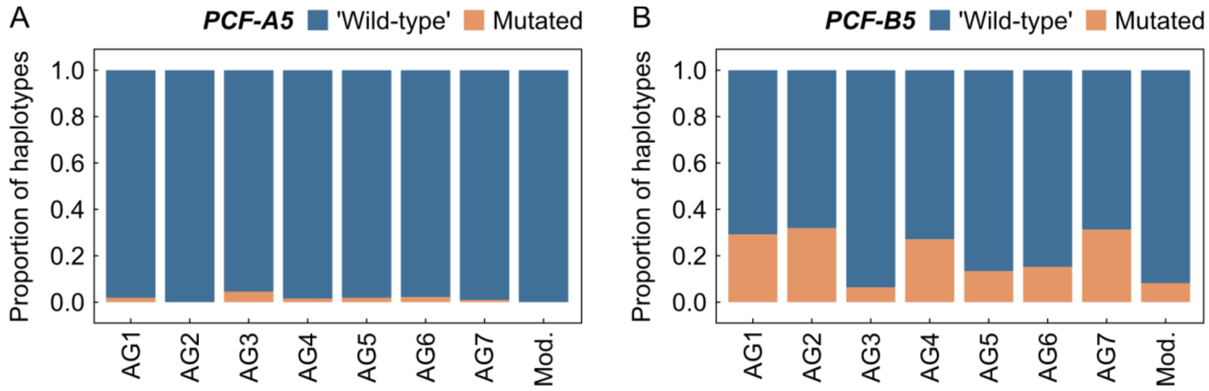

Supplementary Figure S9. Allelic diversity of *PCF5* in the A and B subgenomes

Stacked bar charts showing *PCF-A5* (**A**) and *PCF-B5* (**B**) haplotypes according to representation in ancestral groups (AG) and modern wheat (Mod.)

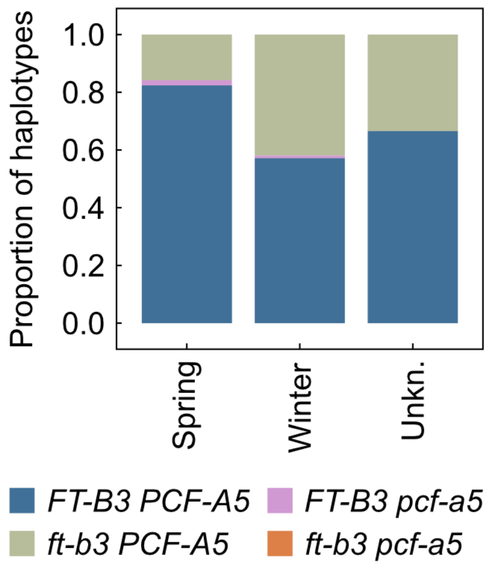

Supplementary Figure S10. Comparison of *PCF-A5* with growth habit and *FT-B3* allele*.* Unkn: Unknown

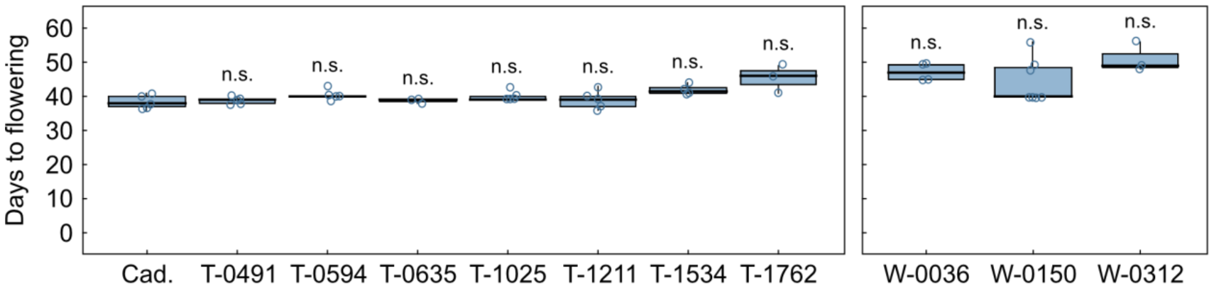

Supplementary Figure S11. Days to flowering for lines with variation in *PCF5* genes

Days to flowering under 22°C ELD of TILLING (T) and Watseq (W) lines with variation in *PCF5* genes, allelic details in **Supplementary Table 5.** Asterisks indicate statistical differences between each genotype and *cv.* Cadenza (Cad.) (Kruskal-Wallis test, Wilcoxon signed-rank test, $p<0.05$, N=3-7), n.s.: not significant.

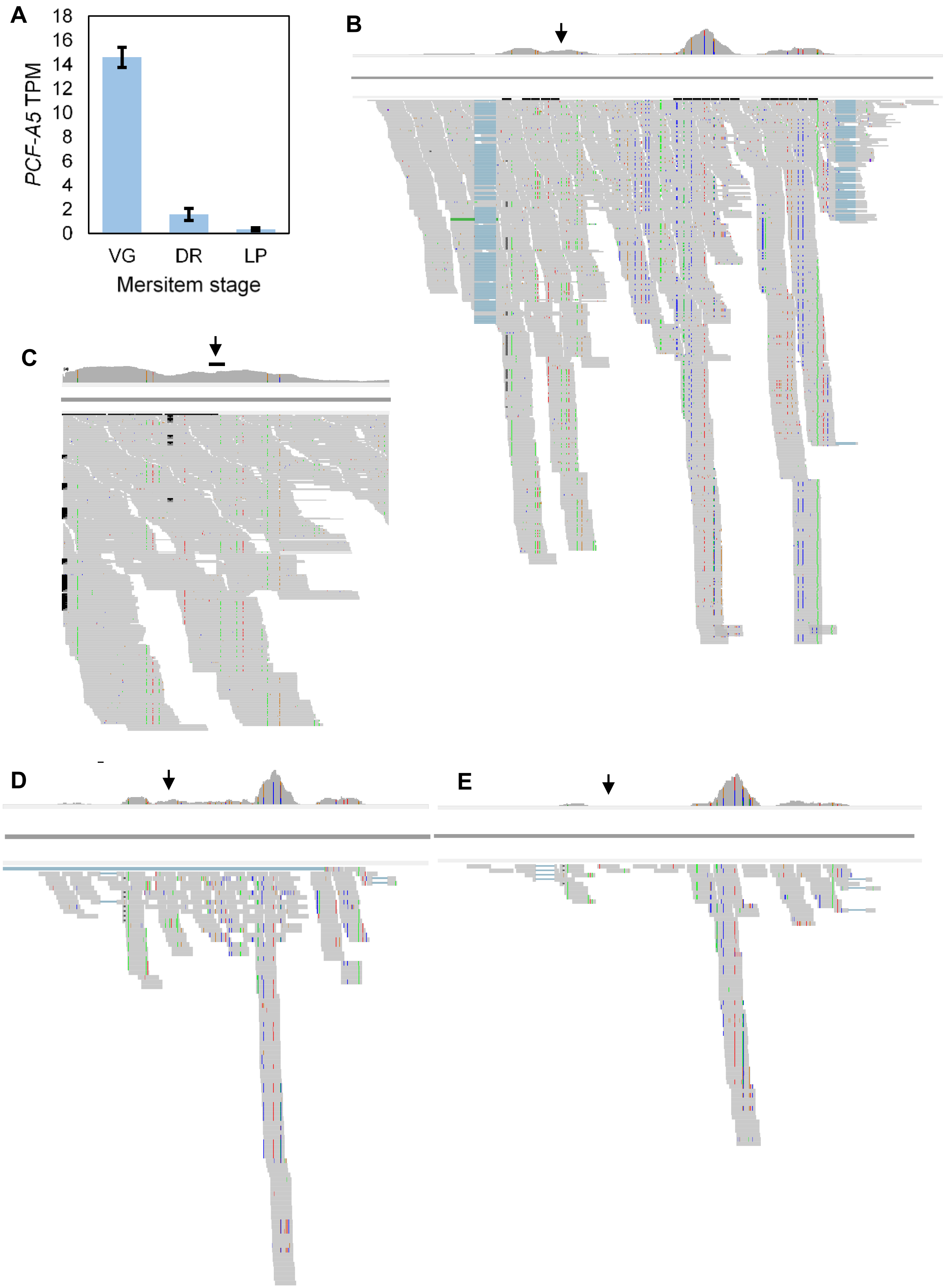

A
18
16
14
12
10
8
6
4
2
0
PCF-A5 TPM
VG
DR
LP
Mersitem stage
B
C
D
E

Supplementary Figure S12. *PCF-A5* expression in the developing floral inflorescence

Transcript profile of *PCF-A5* in the developing floral inflorescence in c.v. Paragon. The defined stages are vegetative (VG), double-ridge (DR), lemma primordium (LP). Data are presented as bar plots that show transcript levels (TPM) ± SEM (error bars) of three biological replicates (**A**). Transcripts aligned to reference genome using IGV (Robinson 2011) to location 3A:119,137,149-119,140,425. MiR319 binding site indicated by a black arrow (**B-E**). Transcripts aligned to VG stage (**B**). Enhanced view of MiR319 binding site for VG stage (**C**). Transcripts aligned to DR stage (**D**). Transcripts aligned to LP stage (**E**). RNA-sequencing data obtained from (Gauley et al. 2024).

SUPPLEMENTARY TABLES

Supplementary Table 1. Genomic variation in FTB3 haplotypes relative to the reference genome Chinese spring.

Supplementary Table 2. Haplotype primers for FT3

Supplementary Table 3. Primers used in this study

Supplementary Table 4. Germplasm used in this study

Supplementary Table 5. Genomic variation in PCF5 haplotypes relative to the reference genome Chinese spring.

References for Supplementary Information

Gauley A, Pasquariello M, Yoshikawa GV, Alabdullah AK, Hayta S, Smedley MA, Dixon LE, Boden SA. 2024. Photoperiod-1 regulates the wheat inflorescence transcriptome to influence spikelet architecture and flowering time. *Curr Biol* **34**: 2330-2343 e2334.

Robinson JT, Thorvaldsdóttir, H., Winckler, W., Guttman, M., Lander, E. S., Getz, G., and Mesirov, J. P. 2011. Integrative genomics viewer. *Nature Biotechnology* **29**.
